# Signaling bias of the protease-activated receptor-1 is dictated by distinct GRK5 and β-arrestin-2 determinants

**DOI:** 10.1016/j.celrep.2026.117041

**Published:** 2026-03-03

**Authors:** Monica L. Gonzalez Ramirez, Lennis B. Orduña-Castillo, Carolyne Bardeleben, Huaping Qin, Ying Lin, Cierra A. Birch, Irina Kufareva, JoAnn Trejo

**Affiliations:** 1 Department of Pharmacology, School of Medicine, University of California, San Diego, La Jolla, CA 92093, USA; 2 Skaggs School of Pharmacy and Pharmaceutical Sciences, University of California, San Diego, La Jolla, CA 92093, USA; 3 These authors contributed equally; 4 Lead contact

## Abstract

G protein-coupled receptors (GPCRs) exhibit signaling bias or preferential activation of heterotrimeric G proteins versus GPCR kinase (GRK)-mediated β-arrestin signaling. The protease-activated receptor-1 (PAR1) activates both G protein and β-arrestin in response to thrombin but only β-arrestin in response to activated protein C (APC). Thrombin-activated PAR1-G protein signaling is desensitized by β-arrestin-1, whereas APC-activated PAR1 signaling is propagated by β-arrestin-2. The mechanisms underlying PAR1 biased signaling are not known. Here, using computational modeling combined with cellular and biochemical studies, we reveal the molecular basis of signaling by thrombin- and APC-activated PAR1. Although both thrombin- and APC-induced PAR1 signaling are regulated by the same GRK, GRK5, the two types of signaling are differentially dependent on GRK5 membrane anchoring, PAR1 C-terminal phosphorylation sites, and the binding mode of β-arrestin-2. These differences translate into distinct β-arrestin-2 conformations and define the APC cytoprotective signaling signature, which contrasts with thrombin inflammatory signaling.

## In brief

Activation of PAR1 by thrombin triggers G protein-induced endothelial dysfunction, whereas APC-activated PAR1 promotes **β**-arrestin-mediated endothelial cytoprotection. This work integrated computational, biochemical, and cellular studies to reveal the basis of thrombin versus APC signaling bias, which is critical for developing drugs that strengthen endothelial cell resilience and resistance to inflammation.

## Graphical Abstract

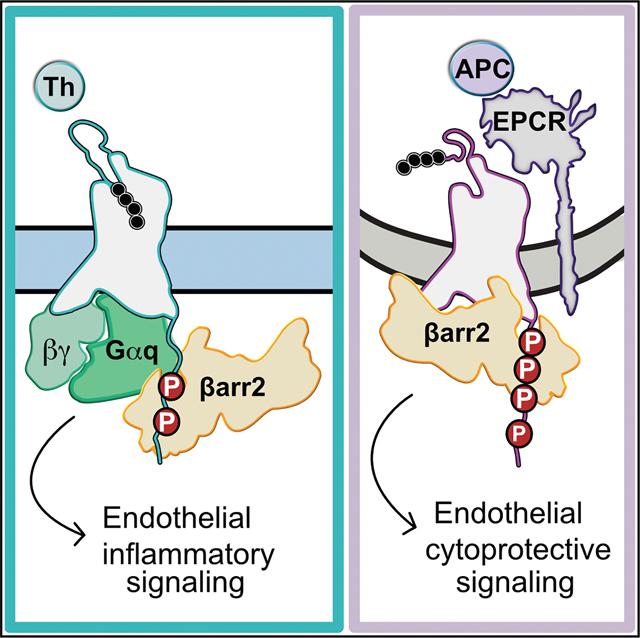

## INTRODUCTION

G protein-coupled receptors (GPCRs) transduce signals in response to a wide range of stimuli, modulate most physiological functions, and are highly druggable. Upon agonist binding, GPCRs undergo conformational changes that facilitate activation of heterotrimeric G protein signaling. Agonist-activated GPCRs are phosphorylated by GPCR kinases (GRKs), which enables β-arrestin (βarr) recruitment. βarrs desensitize agonist-activated GPCR-G protein signaling and function as scaffolds that assemble and activate new signaling complexes. GPCRs display bias toward either G protein- or GRK-mediated βarr signaling depending on the activating agonist.^1,2^ However, the mechanisms that specify how biased agonists induce GPCR preferential activation of heterotrimeric G proteins versus βarr-mediated signaling are complex and not well understood.

Protease-activated receptor-1 (PAR1) is an important drug target^3^ and a GPCR for the coagulant protease thrombin^4^ and anti-coagulant protease activated protein C (APC).^5^ Thrombin activation of PAR1 promotes G protein signaling and mediates platelet activation leading to thrombosis,^6,7^ cancer progression,^8^ and endothelial inflammatory responses, including p38 mitogen-activated protein kinase (MAPK) signaling.^9,10^ The PAR1 antagonist vorapaxar is approved for thrombotic cardiovascular events.^7^ In contrast to thrombin, APC-activated PAR1 promotes βarr2-mediated cytoprotective responses, including endothelial barrier stabilization, anti-apoptotic Akt pro-survival activities,^11,12^ and βarr2-dependent neuroprotection in a mouse model,^13^ making APC variants or synthetic ligands promising therapeutics. Thrombin and APC biased agonism is mediated by differential cleavage of the PAR1 N terminus, resulting in the generation of distinct tethered N termini. Thrombin cleaves PAR1 at the N-terminal arginine (R)-41 site, generating a tethered ligand that triggers heterotrimeric G protein signaling^6^ and is rapidly terminated by βarr1.^14,15^ In contrast, APC bound to the endothelial protein C receptor (EPCR), a transmembrane (TM) cofactor, cleaves PAR1 at the N-terminal R-46 site, generating a different N terminus that activates βarr2-mediated cytoprotective signaling.^11,12,16^

Structural studies indicate that the adoption of distinct conformational states by GPCRs, GRKs, and βarrs is critical for specifying biased signaling.^1,17,18^ The patterns or barcodes of phosphorylation sites on GPCRs induced by different GRKs in response to biased agonists are known to differently modulate βarr function.^17,19,20^ The GRK subtypes 2, 3, 5, and 6 are widely expressed; however, the mechanisms that specify which individual GRK subtype regulates specific GPCR-stimulated βarr functions in response to biased agonists are not well understood. Thus, we sought to understand the molecular basis by which GRKs and βarrs drive PAR1 biased signaling.

In this study, we report that thrombin- and APC-mediated activation of PAR1 promotes distinct conformational states of the receptor TM domain. However, thrombin- and APC-induced PAR1 signaling are both regulated by the same GRK, GRK5. Despite this, the two types of PAR1 biased signaling rely on different GRK5 and βarr2 determinants, distinct receptor C-terminal phosphorylation patterns, and different modes of βarr2 engagement. This translates into distinct conformations of βarr2 that may enable thrombin-activated (Th-activated) PAR1 inflammatory signaling versus APC-activated PAR1 cytoprotective signaling.

## RESULTS

### Structural predictions of PAR1 conformational states induced by biased agonists

**Figure 1.**
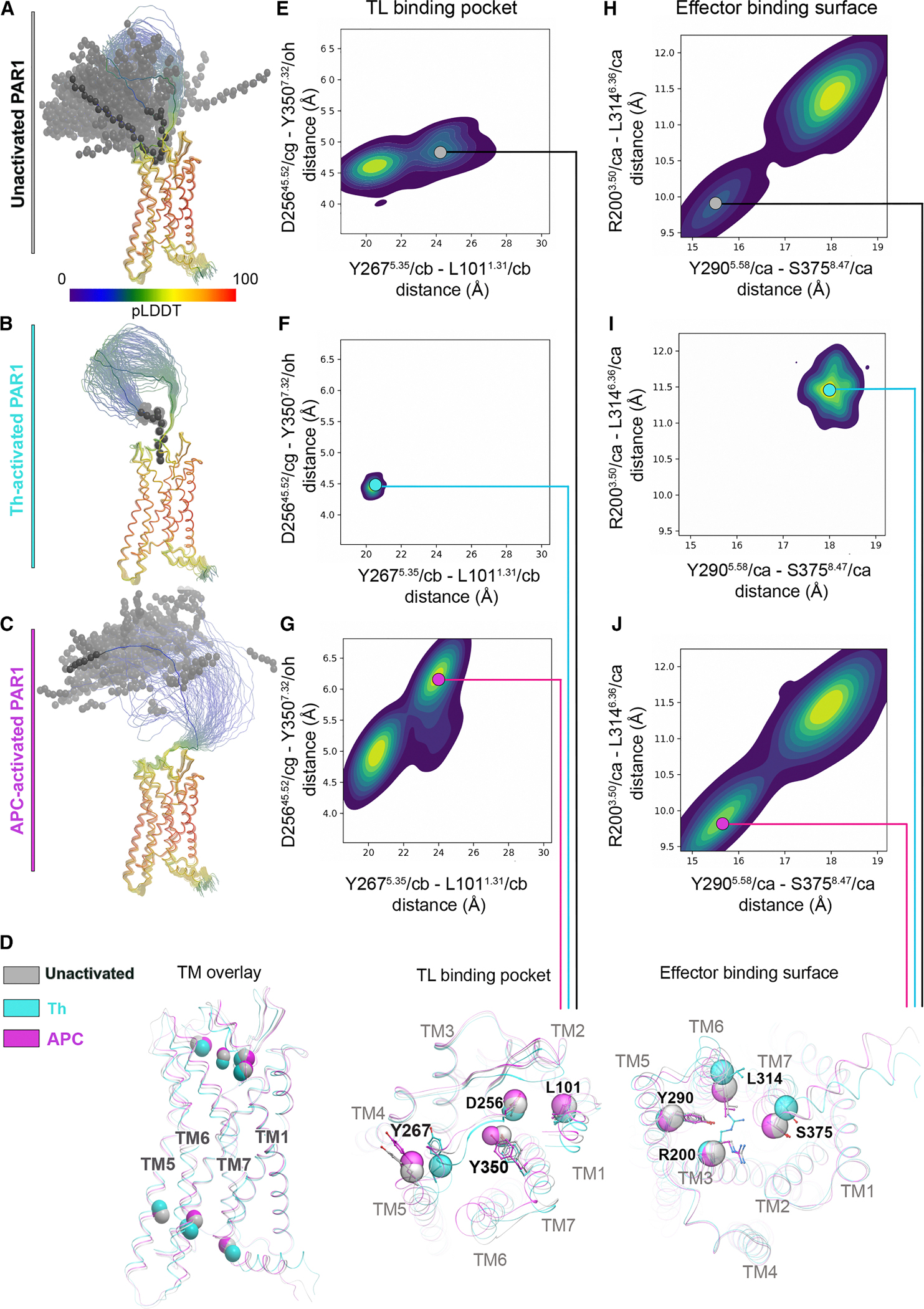
Predicted structures and conformational preferences of unactivated, Th-activated, and APC-activated PAR1 (A–C) AF3 model ensembles of the indicated PAR1 species. Receptors are shown as ribbons (transparent for the entire ensemble, solid for a selected representative conformation) colored by the per-residue predicted local distance difference test (pLDDT) score (reflecting prediction confidence). PAR1 residues 22–52 (A), 42–52 (B), and 47–52 (C) are shown as black spheres. (D) Overlay of representative models for unactivated, thrombin-activated, and APC-activated PAR1 transmembrane (TM) domains. Spheres denote atoms used for calculating intramolecular distances in (E–J). Overlaid TM bundles are viewed parallel to the plane of the membrane (left) or perpendicular to it from the extracellular (middle) or intracellular (right) side. (E–J) The distributions, across the model ensemble, of distances (Å) between key residues in the orthosteric tethered ligand (TL) binding pocket (E–G) and the effector binding surface (H–J). Plots represent unactivated (E and H), thrombin-activated (F and I), and APC-activated (G and J) PAR1. Solid circles indicate representative conformations that are specific to the different species and are shown in (D).

The crystal structure of PAR1 bound to the antagonist vorapaxar^21^ and recent structures of activated PAR1 bound to the thrombin-generated tethered activating ligand have been reported.^22,23^ However, the structure of APC-activated PAR1 is not known. To gain insight into the conformational preferences of unactivated PAR1, Th-activated PAR1, and APC-activated PAR1, we built an ensemble of 100 structural models of each of these molecular species using AlphaFold 3 (AF3).^24^ To reduce conformational bias, all models were built without intracellular effectors or the disordered C terminus and included amino acid residues 22–390, 42–390, and 47–390 for unactivated PAR1, Th-activated PAR1, and APC-activated PAR1, respectively. The model ensembles highlighted clear conformational differences (Figures 1A–1C). Th-activated PAR1 showed high conformational consistency across the ensemble, with the N-terminal tethered ligand docking into the shallow and narrow orthosteric binding site and interacting with key residues important for activation (Figure 1B), as recently reported.^22,23^ In contrast, the ensembles of the unactivated PAR1 and APC-activated PAR1 displayed substantial conformational variability (Figures 1A and 1C). The full-length N terminus of uncleaved PAR1 was predicted to partially dock in the orthosteric pocket in most models and stay out in the solvent in others (Figure 1A). The APC-cleaved N terminus invariably stayed out of the pocket in all 100 models (Figure 1C).

To understand differences in the preferred conformations of the PAR1 TM helical bundle, corresponding to differentially cleaved receptor species, we measured distances between key residues in the PAR1 tethered ligand binding pocket (D256^45.52^/cg-Y350^7.32^/oh and Y267^5.35^/cb-L101^1.31^/cb) and the effector binding surface (R200^3.50^/ca-L314^6.36^/ca and Y290^5.58^/ca-S375^8.47^/ca). In contrast to unactivated PAR1 (Figures 1D and 1E), the Th-activated PAR1 distance map exhibited a constricted tethered ligand binding pocket with shorter distances between the key residue pairs D256-Y350 and Y267-L101 (Figures 1D and 1F) and a uniform extended effector binding surface with longer distances between key residues R200-L314 and Y290-S375 (Figures 1D and 1I). Similar to other class A GPCRs,^25^ this signature corresponds to an active state of PAR1 that favors coupling to heterotrimeric G proteins. By contrast, the orthosteric binding pocket distance maps in the APC-activated PAR1 demonstrated more variable and frequently more open ligand binding pocket conformations (Figures 1D and 1G) compared to Th-activated PAR1 (Figures 1D and 1F) and unactivated PAR1 (Figures 1D and 1E). Consistent with the idea of allosteric communication between the extracellular orthosteric and the intracellular effector-binding pockets, APC-activated PAR1 preferentially featured a narrow effector binding surface (Figures 1D and 1J), which was not observed in Th-activated PAR1 and was only rarely observed in unactivated PAR1 (Figures 1H and 1I). The narrow effector binding interface in APC-activated PAR1 makes it incompatible with the canonical G protein coupling geometry and explains the experimentally observed bias of APC-activated PAR1 toward βarr2.^11–13^ Together, these modeling predictions suggest that thrombin- and APC-activated PAR1 display distinct active conformational states that likely mediate the balanced PAR1-G protein signaling induced by thrombin and βarr2 biased signaling promoted by APC.

### A dual role for GRK5 in the regulation of PAR1 signaling bias

**Figure 2.**
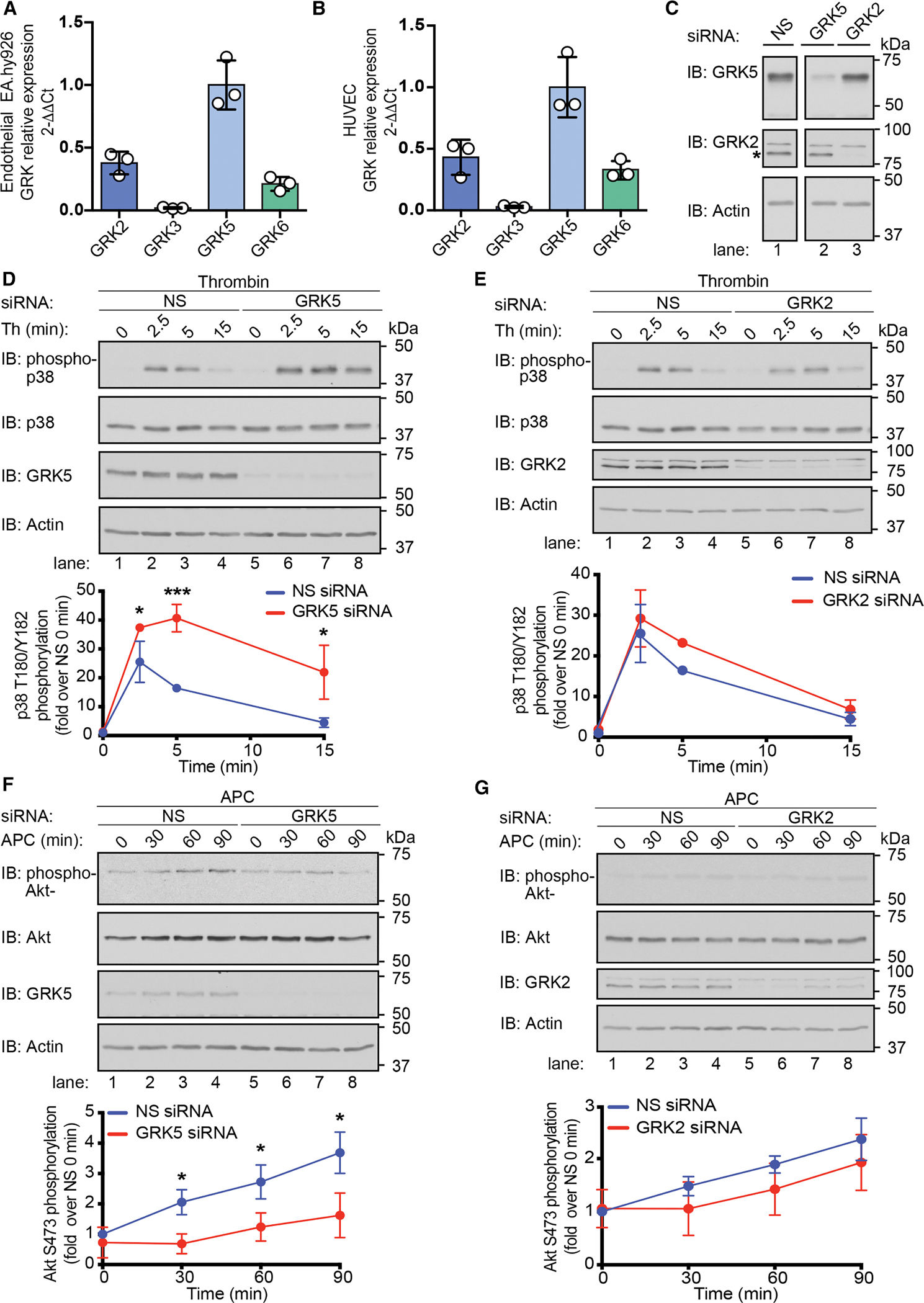
GRK5 regulates both thrombin and APC biased signaling (A and B) RT-qPCR quantified GRK2, GRK3, GRK5, and GRK6 mRNA transcript abundance in HUVEC-derived endothelial EAhy.926 cells (A) and HUVECs (B). Data: mean ± SD, *n* = 3. (C) EA.hy926 cells transfected with the indicated siRNA were immunoblotted for GRK2, GRK5, and actin. NS, non-specific. (D–G) EA.hy926 cells transfected with NS or GRK5 siRNA (D and F), or NS or GRK2 siRNA (E and G), were incubated with 10 nM thrombin or 20 nM APC for various times. Cell lysates were immunoblotted as indicated. Data: mean ± SD, *n* = 3. Statistical significance was determined by a two-way ANOVA followed by Šídák’s multiple comparisons test (*p < 0.05 and ***p < 0.001).

To delineate the function of GRKs in PAR1 biased signaling, we profiled the expression of GRK2, GRK3, GRK5, and GRK6 mRNA transcripts in human cultured endothelial cells using RT-qPCR. In human umbilical vein endothelial cells (HUVEC)-derived EA.hy926 cells, GRK5 showed the highest abundance, whereas GRK2 and GRK6 mRNA transcript expression were detected at ~50% and ~25% of GRK5 mRNA, respectively (Figure 2A). Expression of GRK3 mRNA transcripts was nearly negligible in endothelial EA.hy926 cells (Figure 2A). A similar profile of GRK2, GRK3, GRK5, and GRK6 expression was detected in primary HUVECs (Figure 2B), indicating that GRK5 and GRK2 are the most abundant GRKs expressed in human endothelial cells and are more likely to play a role in PAR1 biased signaling.

A small interfering RNA (siRNA) knockdown approach was next used to directly assess GRK5 and GRK2 function in PAR1 biased signaling in endothelial cells. In cells transfected with non-specific siRNA, GRK5 was detected as an ~65 kDa protein, and GRK2 migrated as an ~80 kDa species (Figure 2C, lane 1). The GRK5-specific siRNA markedly decreased expression of endogenous GRK5 but not GRK2 (Figure 2C, lanes 2 versus 3), whereas the GRK2-specific siRNA virtually abolished GRK2 expression without altering GRK5 expression (Figure 2C, lanes 3 versus 2). To assess the effect of GRK5 and GRK2 knockdown on thrombin signaling, we examined p38 MAPK activation, which is mediated by G protein signaling downstream of activated PAR1.^26^ In endothelial cells transfected with non-specific siRNA, thrombin induced a marked increase in p38 MAPK T180/Y182 phosphorylation at 2.5 min that declined by 15 min (Figure 2D, lanes 1–4). In GRK5-depleted endothelial cells, p38 phosphorylation was significantly increased and prolonged after thrombin treatment compared to control cells (Figure 2D), consistent with a defect in activated PAR1 desensitization. In contrast, the knockdown of GRK2 expression failed to alter thrombin-stimulated p38 phosphorylation compared to non-specific siRNA-transfected cells (Figure 2E, lanes 1–8).

To determine the function of GRK5 and GRK2 in the biased signaling of APC-activated PAR1, we examined Akt pro-survival signaling, which is mediated by βarr2 in endothelial cells.^12,27^ In non-specific siRNA-transfected cells, APC induced a significant increase in Akt S473 phosphorylation at 30 min that remained elevated for 90 min (Figure 2F, lanes 1–4), consistent with previous reports.^12^ However, in GRK5-depleted cells, APC-stimulated Akt signaling was virtually abolished (Figure 2F, lanes 5–8), indicating a critical role for GRK5 in propagating APC-induced Akt signaling. Depletion of GRK2 expression by siRNA had no effect on APC-stimulated Akt signaling compared to control cells (Figure 2G, lanes 1–8). These data reveal a critical role for GRK5 in regulating both thrombin- and APC-activated PAR1 signaling.

### GRK5 is required for both thrombin- and APC-induced βarr2 recruitment

To further interrogate GRK5 function in PAR1 signaling and bias, we optimized a bioluminescence resonance energy transfer (BRET) assay in HEK293 cells to recapitulate thrombin- and APC-induced βarr recruitment. APC requires the EPCR cofactor to facilitate cleavage and activation of PAR1.^16,28^ Therefore, we used HEK293 cells expressing PAR1 fused to yellow fluorescent protein (YFP) and Renilla luciferase (Rluc)-tagged βarr2, with or without EPCR co-expression. Cells were stimulated with thrombin or APC, respectively, and βarr2 recruitment was assessed by BRET (Figure S1A). Thrombin induced a rapid and significant increase in βarr2 association with PAR1, regardless of EPCR expression (Figure S1B), that was completely blocked by vorapaxar (Figure S1B). APC induced a slower hyperbolic and significant increase in PAR1-βarr2 net BRET in cells co-expressing EPCR, which was also abolished by vorapaxar (Figure S1C). In cells lacking EPCR expression, APC-activated PAR1-induced βarr2 recruitment yielded a quasi-linear net BRET increase, suggestive of random collisions rather than specific interactions (Figure S1C) and supporting the notion that EPCR is required for APC efficient activation of PAR1. In addition to kinetic differences, thrombin-induced βarr2 recruitment has a higher amplitude than APC-stimulated βarr2 recruitment in this HEK293 cell system (Figure S1D).

We next examined whether GRK5 expression was required for thrombin- and APC-activated PAR1 recruitment of βarr2 using HEK293 quadruple GRK2,3,5,6 CRISPR-Cas9 knockout (KO) cells^29^ with and without wild-type (WT) GRK5 expression (Figures S1E and S1F). This system provides an effective strategy to dissect the role of GRK5 in PAR1 signaling and bias, avoids potential contributions from other GRKs, and minimizes clone-associated issues.^29,30^ In HEK293 GRK KO cells expressing PAR1-YFP, Rluc-βarr2, and EPCR, no specific increase in PAR1-βarr2 BRET was observed upon thrombin stimulation; however, the BRET signal was fully rescued by re-expression of GRK5 WT (Figures S1F and S1G). Similarly, re-expression of GRK5 WT completely restored APC-activated PAR1-induced βarr2 recruitment (Figures S1F and S1H). Thus, GRK5 expression is sufficient for recruitment of βarr2 to PAR1 following activation with thrombin or APC.

### Distinct GRK5 determinants specify thrombin- versus APC-induced βarr2 recruitment

**Figure 3.**
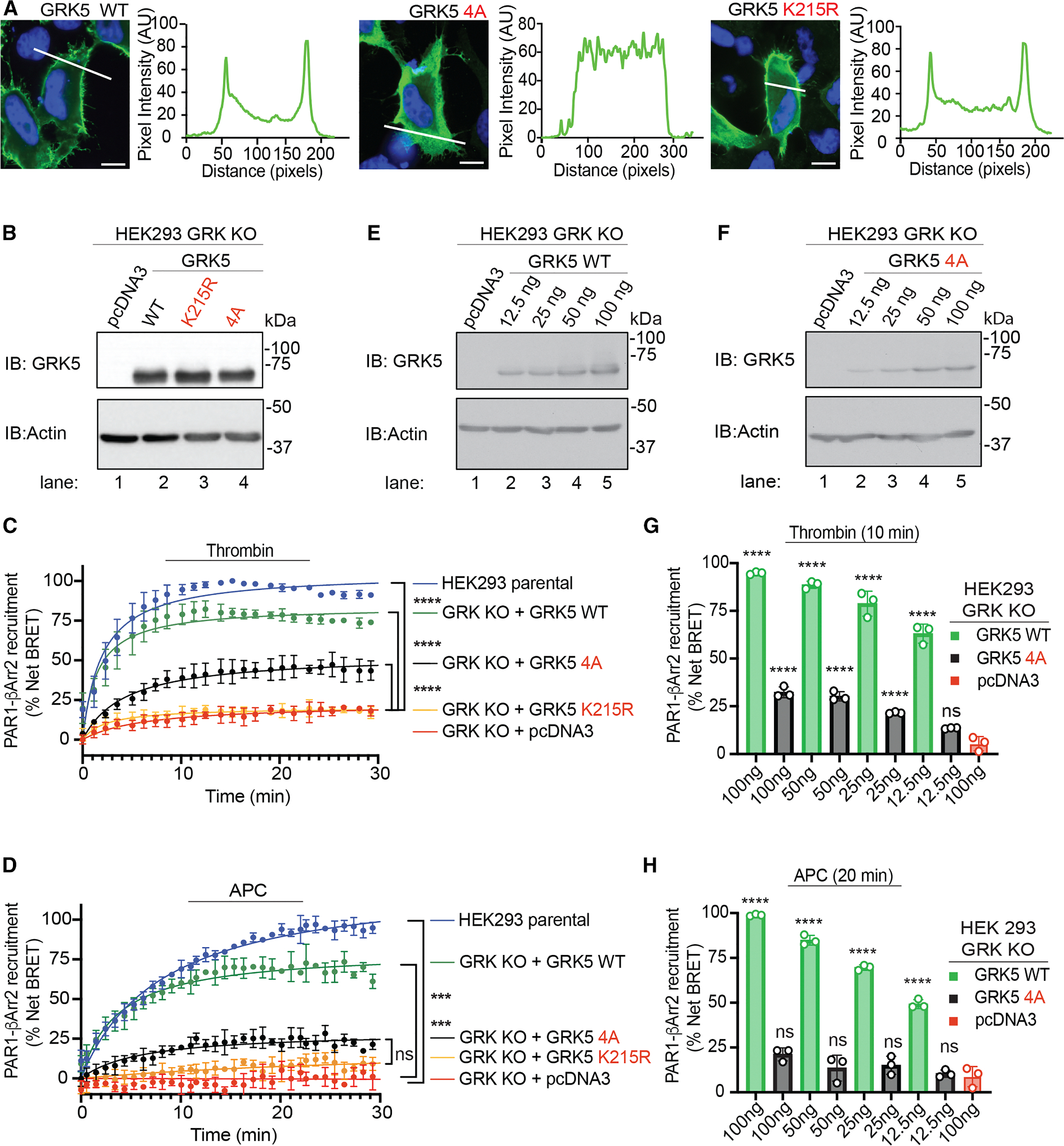
Distinct GRK5 determinants regulate thrombin- versus APC-induced βarr2 recruitment to PAR1 (A) GRK5 WT, 4A, and K215R mutant localization verified by microscopy. The nucleus is stained with DAPI. Scale bar, 100 μm. (B, E, and F) HEK293 GRK KO cells transfected with GRK5 WT, K215R, 4A mutant, or pcDNA3 were immunoblotted as indicated. (C and D) HEK293 parental or GRK KO cells expressing PAR1-YFP, EPCR-Halo, and Rluc-βarr2 with GRK5 WT, K215R, 4A mutant, or pcDNA3 were stimulated with 1 nM thrombin or 20 nM APC and βarr2 recruitment was determined by BRET. Data (mean ± SD, *n* = 3) were analyzed by a one-way ANOVA followed by Tukey’s multiple comparisons test (***p < 0.001, ****p < 0.0001, and ns, not significant). (G and H) HEK293 GRK KO cells transfected with PAR1-YFP, EPCR-Halo, Rluc-βarr2, and GRK5 WT and 4A mutant or pcDNA3 were stimulated with 1 nM thrombin or 20 nM APC, and βarr2 recruitment was determined by BRET. Data (mean ± SD, *n* = 3) were analyzed by a one-way ANOVA followed by Tukey’s multiple comparisons test (****p < 0.0001 and ns, not significant).

GRK5 localizes to the plasma membrane via a C-terminal amphipathic helix, associates with agonist-activated GPCRs, and promotes receptor phosphorylation.^31^ To dissect which specific GRK5 determinants regulate βarr2 recruitment to thrombin- and APC-activated PAR1, we utilized two GRK5 mutants. The first mutant, termed GRK5 4A,^32^ features alanine (A) substitutions for leucine (L550, L551, and L554) and phenylalanine (F555) residues of the amphipathic helix and is defective in plasma membrane localization. The second GRK5 mutant features a lysine (K)215-to-arginine (R) substitution in its active site and is catalytically inactive. The localization of GRK5 WT, 4A, and K215R mutants expressed in HeLa cells was confirmed by immunofluorescence confocal microscopy. Confocal imaging combined with line-scan analysis indicated that GRK5 WT and the catalytically inactive K215R mutant reside primarily at the plasma membrane (Figure 3A), whereas the GRK5 4A mutant redistributed predominantly to the cytoplasm (Figure 3A), as previously reported.^32^

The effect of GRK5 WT and mutants on thrombin- and APC-activated PAR1-induced βarr2 recruitment was next examined using BRET in HEK293 quadruple GRK KO cells. HEK293 GRK KO cells expressing PAR1-YFP, Rluc-βarr2, and EPCR were co-transfected with equivalent amounts of either GRK5 WT, K215R, 4A mutant, or pcDNA3 (Figure 3B). In both thrombin- and APC-stimulated cells, βarr2 recruitment to PAR1 was abolished in GRK KO cells expressing the catalytically inactive mutant K215R or pcDNA3 and restored in cells expressing GRK5 WT, comparable to that observed in HEK293 parental cells (Figures 3C and 3D). Similar to parental cells, thrombin induced a greater increase in βarr2 recruitment compared to APC in HEK293 GRK KO cells expressing GRK5 WT (Figure S2). However, in cells expressing the GRK5 4A mutant at levels comparable to GRK5 WT (Figure 3B), APC/PAR1-induced βarr2 recruitment was negligible and not significant, similar to the catalytically inactive K215R mutant (Figure 3D). This indicates that both GRK5 membrane localization and catalytic activity are critical for βarr2 recruitment to APC-activated PAR1. By contrast, expression of the GRK5 4A mutant resulted in a significant increase in βarr2 recruitment to Th-activated PAR1 (Figure 3C), although such recruitment was still lower than that mediated by GRK5 WT. As expected, the catalytically inactive GRK5 K215R mutant failed to restore thrombin-induced βarr2 recruitment (Figure 3C), indicating that the catalytic activity of GRK5 is essential for βarr2 recruitment to PAR1 in response to both thrombin and APC.

To further examine the impact of GRK5 WT and 4A mutant on PAR1 signaling and bias, we examined the effect of varying GRK5 WT and 4A mutant expression levels on βarr2 recruitment to thrombin- and APC-activated PAR1 by BRET. Compared to βarr2 recruitment induced by Th-activated PAR1 at 10 min in HEK293 GRK KO cells expressing GRK5 WT, the lowest level of GRK5 WT expression allowed for ~60% of βarr2 recruitment signal (Figures 3E and 3G). Similarly, increasing expression of GRK5 WT resulted in a concomitant increase in APC-induced βarr2 recruitment measured at 20 min, with the lowest level of GRK5 WT expression resulting in an ~50% increase in βarr2 recruitment to APC-activated PAR1 (Figures 3E and 3H). GRK5 4A mutant expression resulted in a partial but significant increase in thrombin-stimulated βarr2 recruitment at all except the lowest expression levels (Figures 3F and 3G). In contrast to GRK5 WT, the GRK5 4A mutant failed to significantly enhance APC-induced βarr2 recruitment even at the highest level of expression (Figures 3F and 3H). Thus, unlike thrombin, APC-induced βarr2 recruitment is critically dependent on GRK5 membrane localization for βarr2 recruitment.

### Thrombin and APC require distinct PAR1 C-terminal phosphorylation sites for GRK5-dependent βarr2 recruitment

**Figure 4.**
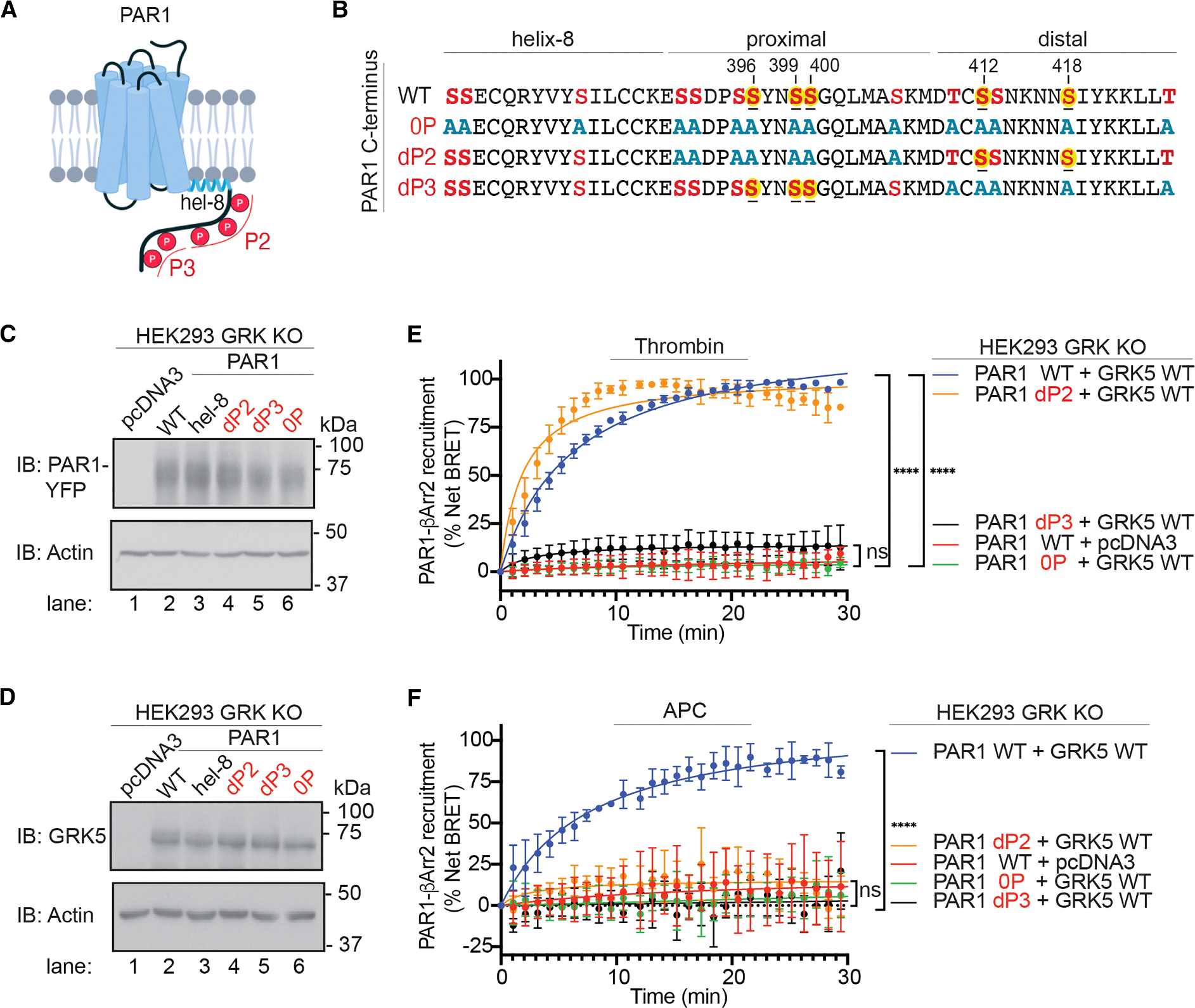
Thrombin- versus APC-induced βarr2 recruitment requires distinct PAR1 C-terminal phosphorylation sites (A) Cartoon of PAR1 C-terminal phosphorylation sites. (B) C-terminal sequences of PAR1 WT, 0P, dP2, and dP3 mutants; known phosphorylation sites are shaded in yellow. (C and D) HEK293 GRK KO cells transfected with PAR1 WT, 0P, dP2, or dP3 mutants with GRK5 WT or pcDNA3 were immunoblotted as indicated. (E and F) HEK293 GRK KO cells transfected with PAR1-YFP WT or mutants, EPCR-Halo, Rluc-βarr2, and GRK5 WT or pcDNA3 were stimulated with 1 nM thrombin or 20 nM APC, and βarr2 recruitment was determined by BRET. Data (mean ± SD, *n* = 3) were analyzed by one-way ANOVA and Tukey’s multiple comparisons test (****p < 0.0001 and ns, not significant).

GPCR biased signaling is driven in part by distinct patterns of GRK-mediated phosphorylation of the receptor C terminus. The C-terminal sequence of PAR1 contains five serine residues, pS396, pS399, pS400, pS412, and pS418, that were previously shown to be phosphorylated by mass spectrometry (Figures 4A and 4B).^33^ A number of additional Ser and Thr phosphorylation sites in the C terminus have also been reported.^33^ To determine if different PAR1 phosphorylation sites are required for thrombin- and APC-induced βarr2 recruitment, three PAR1 C-terminal mutants were generated. First was a fully phospho-deficient PAR1 0P mutant where all serine (S) and threonine (T) residues within the receptor’s helix 8 and the C terminus were mutated to alanine (A). In the dP2 mutant, all candidate Ser/Thr phosphorylation sites in the proximal C terminus were mutated to alanine, while those in the distal C terminus were retained. In the dP3 mutant, candidate Ser/Thr phosphorylation sites in the distal C terminus were mutated to alanine, while those in the proximal C terminus were retained (Figures 4A and 4B).

To examine whether phosphorylation is required for GRK5-dependent βarr2 recruitment to activated PAR1, we used BRET assays. HEK293 GRK KO cells expressing PAR1 WT or the phospho-deficient PAR1 0P mutant fused to YFP (Figure 4C) together with Rluc-βarr2, EPCR, and either GRK5 WT or pcDNA3 (Figure 4D) were stimulated with thrombin or APC, and PAR1-βarr2 BRET was determined. Expression of GRK5 WT rescued βarr2 recruitment to thrombin- and APC-activated PAR1 WT but not to the PAR1 0P mutant (Figures 4E and 4F), indicating that PAR1 C-terminal phosphorylation mediated by GRK5 is required for both thrombin- and APC-induced βarr2 recruitment. Next, we examined whether specific PAR1 C-terminal phosphorylation sites are required for βarr2 recruitment to thrombin- or APC-activated PAR1 using the PAR1 proximal (dP2) and distal (dP3) phospho-site mutants, which expressed at the cell surface equivalent to PAR1 WT and 0P mutant (Figures 4B, 4C, and S3). Thrombin-induced βarr2 recruitment was abolished in cells expressing the PAR1 distal dP3 phospho-site mutant (Figure 4E) and retained in cells expressing the proximal dP2 phospho-site mutant (Figure 4E), indicating that the distal phospho-sites are critical for thrombin-induced βarr2 recruitment. Similar results were observed in HEK293 parental cells, where thrombin-induced βarr2 recruitment was substantially reduced in cells expressing the PAR1 distal dP3 mutant but not in the proximal dP2 phospho-site mutant (Figures S4A and S4C). In contrast to thrombin, both dP2 and dP3 PAR1 phospho-site mutants failed to recruit βarr2 in response to APC (Figure 4F), indicating that both proximal and distal sites of phosphorylation are required for βarr2 recruitment to APC-stimulated PAR1. APC-stimulated βarr2 recruitment was also markedly inhibited in HEK293 parental cells expressing the PAR1 0P, dP2, and dP3 mutants (Figures S4B and S4C), indicating that the results are not attributable to a GRK KO cell-line-specific effect. These results demonstrate distinct phosphorylation pattern requirements for βarr2 association with thrombin- versus APC-activated PAR1, where βarr2 is robustly recruited to Th-activated PAR1 with only distal C-terminal residues phosphorylated, whereas βarr2 association with APC-activated PAR1 requires phosphorylation of both the proximal and distal regions of the C terminus. This agrees with APC-activated PAR1-induced βarr2 recruitment dependence on GRK5 plasma membrane localization (Figure 3) that likely enables GRK5 access to the Ser/Thr sites in the proximal C terminus of PAR1.

### APC, but not thrombin, promotes core engagement between βarr2 and activated PAR1

**Figure 5.**
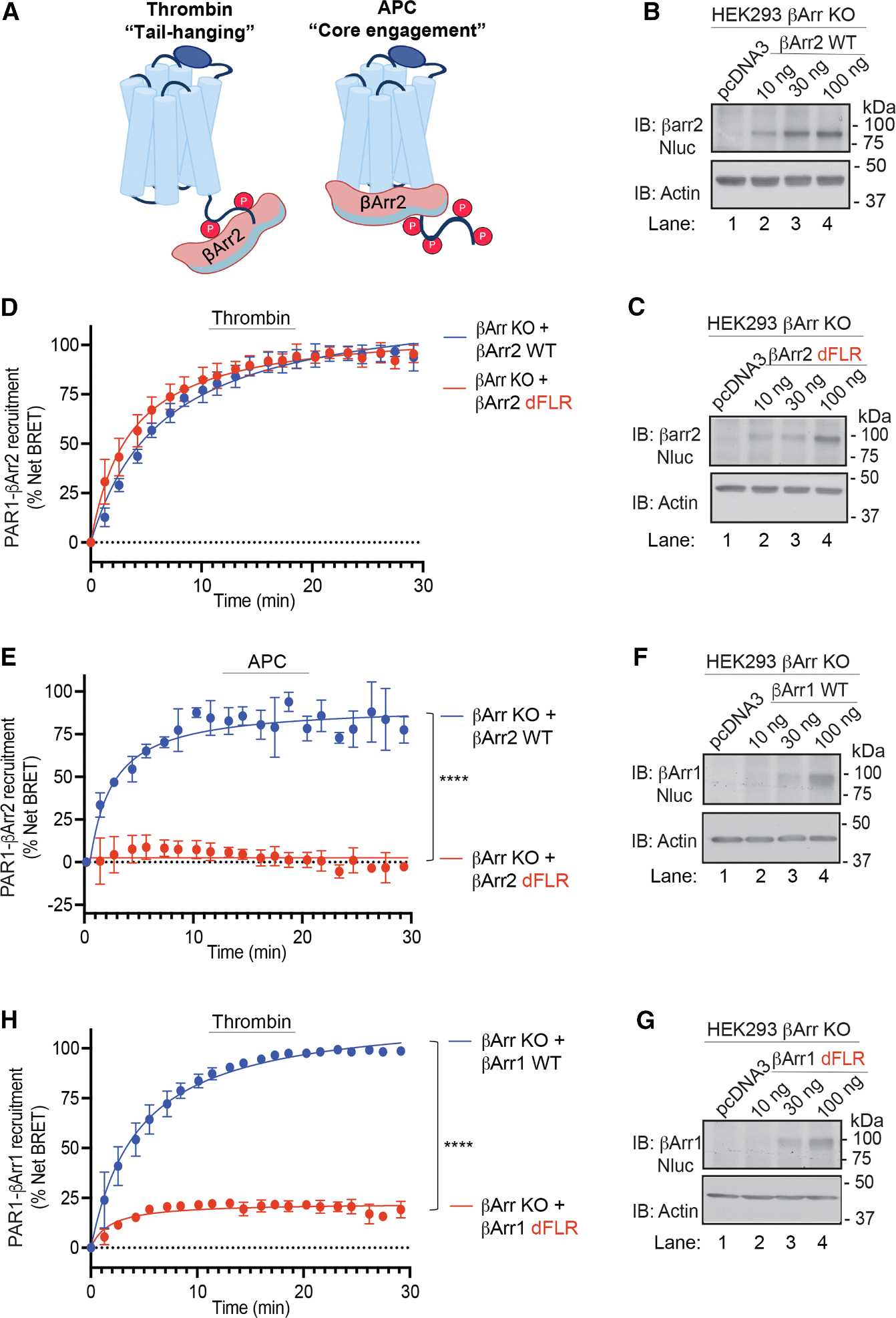
Distinct βarr2 determinants are required for thrombin- versus APC-induced βarr2 recruitment to PAR1 (A) Cartoon of GPCR-βarr2 tail-hanging and core engagement modes. (B, C, F, and G) HEK293 βarr KO cells transfected with PAR1-YFP, EPCR-Halo, and pcDNA3, Nluc-βarr2-WT or -dFLR, or Nluc-βarr1-WT or -dFLR mutant were immunoblotted as indicated. (D and E) HEK293 KO cells transfected with PAR1-YFP, EPCR-Halo, and 100 ng of pcDNA3 or Nluc-βarr2 WT or -dFLR mutant were stimulated with 1 nM thrombin or 20 nM APC, and βarr2 recruitment was determined by BRET. Data (mean ± SD, *n* = 3) were analyzed by Student’s *t* test (****p < 0.0001). (H) HEK293 KO cells transfected with PAR1-YFP, EPCR-Halo, and 100 ng pcDNA3 or Nluc-βarr1 WT or -dFLR were stimulated 1 nM thrombin, and βarr1 recruitment was determined by BRET. Data (mean ± SD, *n* = 3) were analyzed by Student’s *t* test (****p < 0.0001).

βarrs bind to activated GPCRs through at least two distinct modes: one where the finger loop region (FLR) of βarr inserts into the receptor TM core^34–36^ (the core-engaged configuration, Figure 5A) and another exclusively mediated by the receptor’s phosphorylated C terminus (tail-hanging configuration, Figure 5A). To experimentally determine how thrombin- and APC-activated PAR1 engage with βarr2, we compared the recruitment of βarr2 WT to a mutant lacking the FLR (dFLR) using BRET assays. HEK293 βarr1,2 CRISPR-Cas9 KO cells^37^ expressing PAR1-YFP, EPCR, and similar amounts of Nanoluciferase (Nluc)-tagged βarr2 WT or βarr2-dFLR mutant (Figures 5B and 5C) were stimulated with thrombin or APC, and the change in PAR1-βarr2 BRET was determined. Thrombin stimulated a significant increase in both βarr2 WT and dFLR mutant recruitment to activated PAR1 (Figures 5D and S5), suggesting that the FLR-mediated core engagement is dispensable for their interaction, consistent with a tail-hanging configuration of βarr2. APC induced a robust and significant increase in βarr2 WT recruitment, comparable to thrombin (Figures 5E and S5), but failed to promote recruitment of the βarr2 dFLR mutant (Figures 5E and S5). Since Th-activated PAR1 is desensitized by βarr1,^14,15^ we examined whether the FLR was required for βarr1 recruitment to PAR1 using HEK293 cells expressing Nluc-βarr1-WT or the Nluc-βarr1-dFLR mutant (Figures 5F and 5G). In contrast to βarr2 dFLR, thrombin-induced recruitment of βarr1 dFLR to PAR1 was severely compromised (Figure 5H), indicating that, unlike βarr2, βarr1 engages with the TM core of Th-activated PAR1.

**Figure 6.**
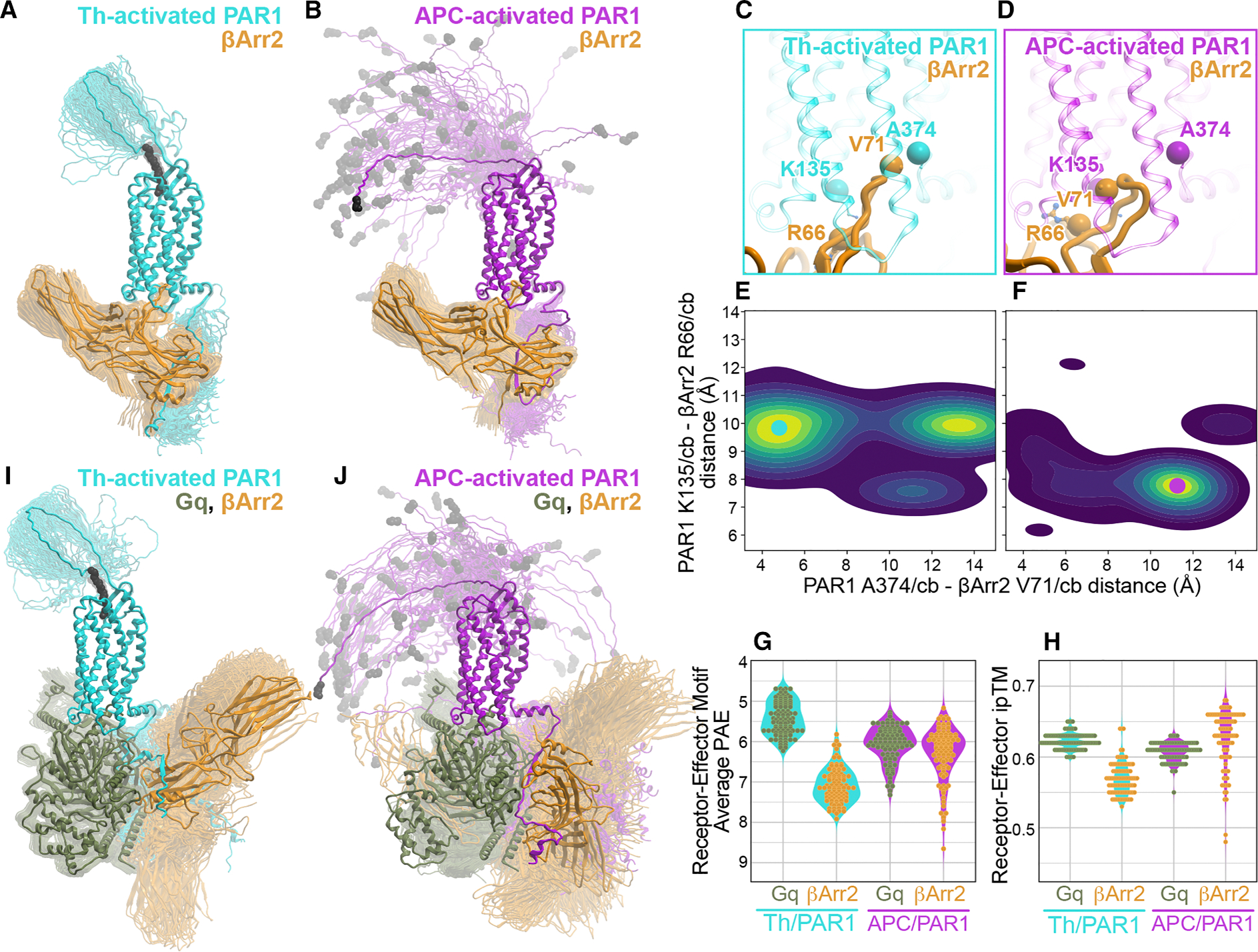
Structure prediction of thrombin- and APC-activated PAR1 bound to βarr2 only or Gq-βarr2 (A, B, I, and J) AF3 models of Th-activated (A and I) or APC-activated (B and J) PAR1 bound to βarr2 only (A and B) or Gq-βarr2 (I and J). Receptors and effectors are shown as ribbons (transparent for the entire ensemble, solid for a selected representative conformation), and PAR1 residues 42–48 (A and I) or 47–48 (B and J) are shown as black spheres. PAR1 C-terminal phospho-sites are not shown. (C and D) The intracellular part of the TM bundle of Th-activated (C) or APC-activated (D) PAR1 with the βarr2 FLR. Receptor and βarr2 are shown as ribbons; spheres denote atoms used to calculate distances in (E) and (F). (E and F) The distributions, across the model ensembles, of distances (Å) between the indicated PAR1 and βarr2 residues. The solid circles indicate representative conformations shown in (C) and (D). (G and H) The distribution of average interface PAE and ipTM scores across the ensembles of Th/PAR1 and APC/PAR1 complexes with Gq-βarr2 or βarr2 alone.

To investigate the structural basis of favorable core-mediated coupling between βarr2 and APC-activated but not Th-activated PAR1, we constructed 100 models of βarr2 complexes with Th-activated PAR1 with distal C-terminus phosphorylated residues (42–425 with pT410, pS412, pS413, and pS418) and APC-activated PAR1 with both distal and proximal C-terminus phosphorylated residues (47–425 with pS391, pS392, pS395, pS396, pS399, pS400, pT410, pS412, pS413, and pS418). When presented with these complex compositions, AF3 failed to predict the tail-hanging mode for βarr2 with Th-activated PAR1 and instead predicted a core-engaged model for all complexes (Figures 6A and 6B). However, the geometry of the finger loop interaction with PAR1 was notably different in APC-activated complexes compared to Th-activated complexes (Figures 6C and 6D). In APC-activated complexes, the preferred finger loop conformation was more compact (Figure 6D), whereas in Th-activated complexes, it was in an extended conformation (Figure 6C). This is also demonstrated by the distances measured between key interaction residues in the PAR1 core region (K135^2.37^/cb and A374^7.56^/cb) and the FLR of βarr2 (R66/cb and V71/cb). In the Th-activated PAR1 ensemble, a substantial population of models has PAR1 A374 and βarr2 V71 in close proximity (Figures 6C and 6E), whereas in the APC-activated model ensemble, these residues are predominantly far apart (Figures 6D and 6F). The predominant population in the APC-activated PAR1 ensemble also features shorter distances between PAR1 K135 and βarr2 R66, compared to the Th-activated ensemble (Figures 6E and 6F). Finally, compared with Th-activated PAR1, AF3 models of βarr2 complexes with APC-activated PAR1 have lower average predicted aligned error (PAE) for βarr2 and higher interface predicted template modeling (ipTM) scores at the βarr2-FLR-PAR1 interface, both indicative of a higher-confidence prediction (Figures 6G and 6H). Thus, despite being unable to predict the tail-hanging mode for βarr2 complex with Th-activated PAR1, AF3 captured the conformational differences in the receptor TM domains caused by differential N-terminal cleavage and C-terminal phosphorylation, and these differences translated into distinct finger loop conformations and varying prediction confidence.

### Structural predictions of thrombin- and APC-activated PAR1 in complex with Gq-βarr2

Our results are consistent with Th-activated but not APC-activated PAR1 forming multimeric complexes that simultaneously include a core-engaged heterotrimeric G protein and a tail-hanging βarr2. To reveal the structural basis for such complexes, we generated 100 models of either thrombin- or APC-activated PAR1 with different phospho-sites as described above (Figures 6A and 6B) in complex with both Gq and βarr2 using AF3 (Figures 6I and 6J). The models of the Th-activated PAR1 Gq-βarr2 complex featured high conformational consistency across the ensemble, with the Gαq subunit C-terminal α helix inserted in the receptor TM core and the βarr2 N domain bound to the distal C-terminal phospho-sites in a tail-hanging mode (Figure 6I). The PAR1-Gq complexes featured low PAE and high ipTM scores for the C-terminal helix of Gαq (Figures 6G and 6H), indicative of high prediction confidence. Notably, AF3 failed to recognize the inability of APC-activated PAR1 to couple to Gq; therefore, it (incorrectly) predicted canonical core-engaged geometry for Gq and a tail-hanging geometry for βarr2 for both thrombin- and APC-activated PAR1 (Figures 6I and 6J). However, the APC-activated PAR1-Gq-βarr2 ensemble was more divergent (Figure 6J) and showed a higher PAE and lower ipTM score for the C-terminal helix of Gαq compared to Th-activated PAR1 (Figures 6G and 6H), indicating lower prediction confidence. We also observed that both the average PAE and ipTM metrics for the C-terminal helix of Gαq in the multimeric complex with Th-activated PAR1 were more favorable than the corresponding metrics in the βarr2 core-engaged complex (Figures 6G and 6H). By contrast, for APC-activated PAR1, the metrics of the βarr2 core-engaged complex indicate higher confidence compared to the multimeric Gq-containing complex (Figures 6G and 6H). These findings are consistent with APC-activated PAR1 preference for βarr2 binding rather than G protein coupling.^11,38^ Altogether, the computational analysis supports our experimental data and demonstrates that thrombin- and APC-activated PAR1 conformations are distinct and enable preferential interactions with G proteins versus βarr2.

### Distinct βarr2 conformational changes induced by thrombin- versus APC-activated PAR1

**Figure 7.**
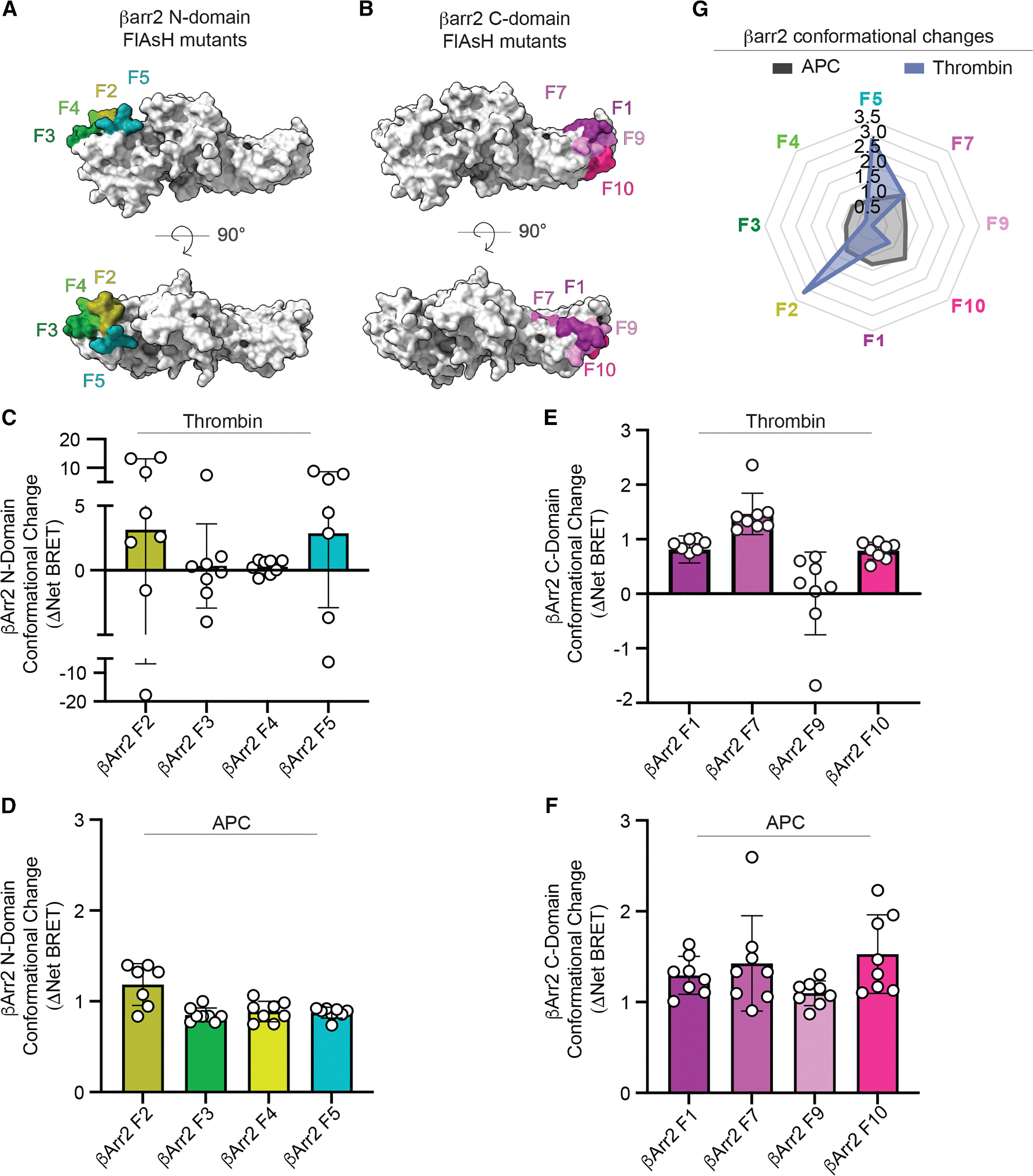
Thrombin and APC induce distinct βarr2 conformational states (A and B) βarr2 inactive structure (PDB: 3P2D) surface projection of the N-domain F2, F3, F4, and F5 and C-domain F1, F7, F9, and F10 FlAsH-binding motifs of the Nluc-βarr2 FlAsH BRET biosensors. (C–F) HEK293 cells transfected with PAR1, EPCR-Halo, and individual Nluc-β-arr2 FlAsH N-domain or C-domain biosensors were stimulated with 1 nM thrombin (C and E) or 20 nM APC (D and F), and BRET was determined. Data: mean ± SD, *n* = 7 or 8. (G) Radar chart of APC- versus thrombin-induced βarr2 conformational changes shown in (C)–(F).

βarrs adopt distinct activation states that are driven in part by the phosphorylation patterns on the C termini of activated GPCRs. To examine whether thrombin and APC induce different βarr2 conformations, we used a series of intramolecular Nluc-βarr2 fluorescein arsenical hairpin (FlAsH)-based BRET biosensors.^39,40^ Each biosensor contains a tetracysteine motif that binds fluorescent arsenical (F) inserted at different positions in the N domain or C domain to monitor conformational changes induced by βarr2 recruitment to agonist-activated GPCRs (Figures 7A and 7B). To compare βarr2 conformational changes induced by thrombin and APC, HEK293 cells were transfected with individual Nluc-βarr2 N-domain F2, F3, F4, or F5 biosensors or with individual Nluc-βarr2 C-domain F1, F7, F9, or F10 biosensors and stimulated with saturating concentrations of thrombin or APC, after which BRET was determined. Thrombin induced robust conformational changes in the βarr2 N-domain F2 and F5 biosensors and minimal conformational changes in the F3 and F4 biosensors (Figure 7C). By contrast, APC promoted modest conformational changes in all the βarr2 N-domain F2, F3, F4, and F5 biosensors (Figure 7D). In studies with the βarr2 C-domain biosensors, thrombin caused a modest change in βarr2 C-domain F1, F7, and F10 biosensors (Figure 7F) but not in the F9 βarr2 biosensor. Similar modest conformational changes in βarr2 C-domain F1, F7, F9, and F10 biosensors were observed with APC (Figure 7F), except for F9, which retained sensitivity to APC stimulation, unlike thrombin (Figures 7F and 7E). These results indicate that thrombin and APC induce distinct βarr2 conformational changes as reflected in the radar chart (Figure 7G). The differences in βarr2 conformations are likely the result of core-versus tail-mediated βarr2 engagement by APC-activated versus Th-activated PAR1 (Figure 5), stemming from differential C-terminal phosphorylation patterns (Figure 4) and consistent with differential GRK5 localization requirements (Figure 3). Altogether, these differences may explain the distinct functional responses (Figure S6), particularly for APC-activated PAR1 βarr2-mediated cytoprotective versus thrombin-induced inflammatory signaling.^11–13^.

## DISCUSSION

The mechanisms that specify which GRK subtype regulates a given GPCR are not clear. Here, we report a unique regulatory mechanism for GPCR biased signaling that is mediated by GRK5. In endothelial cells, we found that GRK5 is the most abundant subtype and functions as the key regulator of both thrombin- and APC-activated PAR1 signaling. GRK5 was previously shown to regulate Th-activated PAR1 desensitization in endothelial cells.^41^ In human platelets, PAR1 and the second thrombin receptor PAR4 are both regulated by GRK5 and GRK6,^42^ although GRK6 is the most abundant subtype.^43^ In addition to expression, the accessibility and conformation of activated PAR1 are likely important for specifying GRK5 subtype function. Unlike GRK2 and GRK3, GRK5 is primarily localized at the plasma membrane via a C-terminal amphipathic helix. We found that a plasma-membrane-deficient GRK5 4A amphipathic helix mutant failed to restore APC-induced βarr2 recruitment, whereas thrombin-induced βarr2 recruitment was partially rescued. Thus, GRK5 membrane localization may increase access to APC-activated PAR1 to enable phosphorylation of proximal and distal sites sufficient for βarr2 recruitment. In contrast, Th-activated PAR1-Gq signaling is likely to induce GRK5 translocation from the plasma membrane to the cytosol through Ca^2+^-mediated calmodulin activation,^44^ thereby limiting access and phosphorylation of PAR1. Unlike Th-activated PAR1 rapid amplification of G protein signaling, APC-activated PAR1-βarr2 signaling onset is slow, prolonged, and dependent on caveolae plasma membrane microdomains^11,38,45^ (Figure S6). GRK5 contains an N-terminal caveolin binding motif, binds caveolin-1, and localizes to caveolae.^46^ Thus, GRK5 membrane localization may also increase accessibility to PAR1 by concentrating these molecules in caveolae.

The barcode hypothesis states that distinct GPCR phosphorylation patterns differentially modulate βarr function. Studies have shown that GRK2/3 and GRK5/6 can phosphorylate the same GPCR at different sites to confer distinct βarr-driven functions such as internalization or signaling.^47–49^ Other studies reported that GPCR biased agonists utilize different GRKs to initiate distinct βarr-mediated responses. The β2-adrenergic receptor (β2AR) biased agonist carvedilol recruits GRK5/6 more efficiently over other GRKs and promotes a different phosphorylation pattern and βarr2-elicited functions.^47,50^ Utilization of GRK5/6 over GRK2/3 by the angiotensin II type-1 receptor (AT1R) biased agonist TRV027 also preferentially induces βarr2-dependent responses.^29^ In contrast to classic biased agonists, we found that GRK5 is utilized by both PAR1 biased agonists, but different C-terminal phosphorylation patterns are required to enable βarr2 recruitment and distinct conformations.

βarrs are known to associate with agonist-activated GPCRs in at least two different configurations: a tail-hanging and a core-engaged mode.^34–36^ Thrombin- and APC-activated PAR1s were found to engage with βarr2 using different modes. APC-induced βarr2 recruitment requires the FLR, indicating core engagement, whereas the FLR is dispensable for thrombin-stimulated βarr2 recruitment, suggesting a tail-hanging mode. The different PAR1-βarr2 binding configurations are consistent with the distinct C-terminal phosphorylation requirements. βarr2 interaction with APC-activated PAR1 requires phosphosites spanning the proximal P2 and distal P3 regions, whereas for Th-activated PAR1, only the distal P3 phospho-sites are necessary. AF3 modeling indicates high confidence for the APC-activated PAR1-βarr2 complex with core engagement and Th-activated PAR1-Gq-βarr2 complex with tail-hanging βarr2 conformation. The tail-hanging βarr2 binding to Th-activated PAR1 is permissive of the formation of Gq- and βarr2-containing “mega-complexes,” but a core-engaged mode for APC-activated PAR1 excludes its simultaneous coupling to G proteins. However, experimental confirmation of the PAR1-Gq-βarr2 mega complexes is challenging, requires isolation of the stable complex and G protein activation *in vitro*,^51^ and has not been obtained.

In contrast to βarr2, βarr1 recruitment induced by Th-activated PAR1 is dependent on the FLR structural element and requires the P3 distal phosphorylation sites of PAR1,^52^ similar to βarr2. Thus, βarr subtypes are not redundant and can engage the same agonist-activated GPCR using similar phosphorylation sites, yet they adopt distinct conformations, a phenomenon that may rely on subtype-specific intrinsic features.

The current paradigm postulates that the GPCR-βarr tail-hanging mode facilitates internalization and G protein signaling, whereas core engagement promotes termination of G protein signaling.^1,53^ We show that thrombin- and APC-activated PAR1 engage with distinct βarr1 and βarr2 configurations and have similar and different functional implications compared to the current paradigm. In fibroblasts derived from βarr KO mice, we showed that βarr1, and not βarr2, is critical for desensitization of Th-activated PAR1 G protein signaling.^14,15^ We further demonstrated that PAR1 internalization is independent of both βarr1 and βarr2 and requires the μ2 adaptin subunit of the clathrin adaptor protein-2 complex to promote clathrin-mediated endocytosis.^54–56^ Thus, Th-activated PAR1-βarr1 core engagement prevents G protein activation but is not required for receptor internalization, consistent with the prevailing paradigm. The function of Th-activated PAR1-βarr2 tail-hanging engagement is not known, but this configuration appears to be permissive of receptor association with other effectors,^34^ including simultaneous G protein coupling,^51,57^ as predicted by AF3 modeling. Although βarr2 is required for APC-activated PAR1 biased signaling in various cell types and mouse models,^11–13^ APC-activated PAR1 fails to internalize from the cell surface.^38,58^ These findings are consistent with an APC-induced PAR1-βarr2 core engagement configuration that occludes G protein activation and elicits βarr2 signaling but is incapable of promoting receptor internalization. Currently, it is not known why the APC-activated βarr2 conformation fails to promote receptor internalization; the phenomenon may be due to the receptor phosphorylation state, caveolae membrane microdomain localization, and microdomain lipid composition or other features inherent to βarr2.

In summary, we report that PAR1 biased signaling is driven by different activated PAR1 conformational states and regulated by the same GRK subtype, GRK5. GRK5 promotes distinct βarr2 activated conformational states induced by biased agonists that are driven by different PAR1 C-terminal phosphorylation patterns. Furthermore, the GRK5 amphipathic helix and βarr2 FLR region, distinct structural elements, mediate the specificity of APC versus thrombin biased signaling. The unique regulatory mechanism for GPCR biased signaling is strongly supported by AF3 computational modeling prediction and experimental validation. One unexpected finding is that APC-activated PAR1 biased agonism appears to occur without the predicted insertion of the cleaved N-terminus into the receptor orthosteric binding pocket. Additional studies are necessary to validate the structural model and to resolve this phenomenon.

### Limitations of the study

The use of protein overexpression, gene KOs, siRNA knockdowns of target proteins, and inhibitor treatments can lead to artifacts and non-specific effects. To mitigate the risks, standard protocols and cell passage conditions, rescue experiments, and orthogonal assays were utilized. AF3 modeling does not predict the existence of a defined complex, as AF3 has been trained on available structures. For example, in this work, AF3 failed to predict tail-binding βarr2 conformations in the absence of G protein (such complexes were only sparsely represented in its training set). Thus, we rely on AF3 to generate hypotheses and to rationalize our experimental findings. AF3 modeling provides a structural context for the experimentally observed phenomena. Its expected accuracy is high and approaches (or may even exceed) that of low-resolution cryo-electron microscopy.^59^ Although molecular dynamics is the most rigorous way to assess PAR1 conformational changes induced by distinct N-terminal tails and C-terminal phosphorylation patterns, AF3 ensemble modeling can provide initial insight into such dynamics through conformational variability,^60,61^ which we exploit in our study.

## RESOURCE AVAILABILITY

### Lead contact

Requests for further information and resources should be directed to and will be fulfilled by the lead contact, JoAnn Trejo (jotrejo@health.ucsd.edu).

### Materials availability

All unique/stable reagents generated in this study are available from the lead contact with a completed materials transfer agreement.

### Data and code availability

- Coordinates of PAR1 AF3 model ensembles have been deposited to Zenodo: https://doi.org/10.5281/zenodo.18816262.
- The rest of the data reported in this paper will be shared by the lead contact upon request.
- This paper does not report original code.
- Any additional information required to reanalyze the data reported in this work paper is available from the lead contact upon request.

## STAR★METHODS

Detailed methods are provided in the online version of this paper and include the following:

### EXPERIMENTAL MODEL AND STUDY PARTICIPANT DETAILS

Cell lines. EA.hy926 cells were authenticated by short tandem repeat profiling. HUVEC were authenticated by expression of CD31/105 and von Willebrand Factor VIII, and assessment of acetylated low-density lipoprotein uptake. HEK293A parental cells, HEK293A CRISPR-Cas9 β-arrestin-1,2 KO cells, and HEK293A GRK 2,3,5,6 KO cells were obtained from Dr. Asuka Inoue (Tohoku University) and verified by restriction enzyme-based genotyping and immunoblot.^37,62^ All cell lines were routinely monitored for mycoplasma using Venor GeM Mycoplasma detection kit (Sigma-Aldrich) per the manufacturer’s instructions.

### METHOD DETAILS

#### Modeling and analysis of PAR1 complexes

AlphaFold 3^24^ server was used to generate three-dimensional models of human PAR1 with varying phosphorylation patterns, with and without effector proteins.

The following amino acid sequences and phosphorylation patterns were used for PAR1 (all based on UniProt entry P25116, PAR1_HUMAN):

- PAR1(22–390) (mature unactivated PAR1 lacking the disordered C terminus): ARTRARRPESKATNATLDPRSFLLRNPNDKYEPFWEDEEKNESGLTEYRLVSINKSSPLQKQLPAFISEDASGYLTSSWLTLFVPSVYTGVFVVSLPLNIMAIVVFILKMKVKKPAVVYMLHLATADVLFVSVLPFKISYYFSGSDWQFGSELCRFVTAAFYCNMYASILLMTVISIDRFLAVVYPMQSLSWRTLGRASFTCLAIWALAIAGVVPLLLKEQTIQVPGLNITTCHDVLNETLLEGYYAYYFSAFSAVFFFVPLIISTVCYVSIIRCLSSSAVANRSKKSRALFLSAAVFCIFIICFGPTNVLLIAHYSFLSHTSTTEAAYFAYLLCVCVSSISCCIDPLIYYYASSECQRYVYSILCCKE
- PAR1(42–390) (thrombin-activated PAR1 lacking the disordered C terminus): SFLLRNPNDKYEPFWEDEEKNESGLTEYRLVSINKSSPLQKQLPAFISEDASGYLTSSWLTLFVPSVYTGVFVVSLPLNIMAIVVFILKMKVKKPAVVYMLHLATADVLFVSVLPFKISYYFSGSDWQFGSELCRFVTAAFYCNMYASILLMTVISIDRFLAVVYPMQSLSWRTLGRASFTCLAIWALAIAGVVPLLLKEQTIQVPGLNITTCHDVLNETLLEGYYAYYFSAFSAVFFFVPLIISTVCYVSIIRCLSSSAVANRSKKSRALFLSAAVFCIFIICFGPTNVLLIAHYSFLSHTSTTEAAYFAYLLCVCVSSISCCIDPLIYYYASSECQRYVYSILCCKE
- PAR1(47–390) (APC-cleaved PAR1 lacking the disordered C terminus): NPNDKYEPFWEDEEKNESGLTEYRLVSINKSSPLQKQLPAFISEDASGYLTSSWLTLFVPSVYTGVFVVSLPLNIMAIVVFILKMKVKKPAVVYMLHLATADVLFVSVLPFKISYYFSGSDWQFGSELCRFVTAAFYCNMYASILLMTVISIDRFLAVVYPMQSLSWRTLGRASFTCLAIWALAIAGVVPLLLKEQTIQVPGLNITTCHDVLNETLLEGYYAYYFSAFSAVFFFVPLIISTVCYVSIIRCLSSSAVANRSKKSRALFLSAAVFCIFIICFGPTNVLLIAHYSFLSHTSTTEAAYFAYLLCVCVSSISCCIDPLIYYYASSECQRYVYSILCCKE
- PAR1(42–425) (full-length thrombin-activated PAR1): SFLLRNPNDKYEPFWEDEEKNESGLTEYRLVSINKSSPLQKQLPAFISEDASGYLTSSWLTLFVPSVYTGVFVVSLPLNIMAIVVFILKMKVKKPAVVYMLHLATADVLFVSVLPFKISYYFSGSDWQFGSELCRFVTAAFYCNMYASILLMTVISIDRFLAVVYPMQSLSWRTLGRASFTCLAIWALAIAGVVPLLLKEQTIQVPGLNITTCHDVLNETLLEGYYAYYFSAFSAVFFFVPLIISTVCYVSIIRCLSSSAVANRSKKSRALFLSAAVFCIFIICFGPTNVLLIAHYSFLSHTSTTEAAYFAYLLCVCVSSISCCIDPLIYYYASSECQRYVYSILCCKESSDPSSYNSSGQLMASKMDTCSSNLNNSIYKKLLT
- PAR1(47–425) (full-length APC-cleaved PAR1): NPNDKYEPFWEDEEKNESGLTEYRLVSINKSSPLQKQLPAFISEDASGYLTSSWLTLFVPSVYTGVFVVSLPLNIMAIVVFILKMKVKKPAVVYMLHLATADVLFVSVLPFKISYYFSGSDWQFGSELCRFVTAAFYCNMYASILLMTVISIDRFLAVVYPMQSLSWRTLGRASFTCLAIWALAIAGVVPLLLKEQTIQVPGLNITTCHDVLNETLLEGYYAYYFSAFSAVFFFVPLIISTVCYVSIIRCLSSSAVANRSKKSRALFLSAAVFCIFIICFGPTNVLLIAHYSFLSHTSTTEAAYFAYLLCVCVSSISCCIDPLIYYYASSECQRYVYSILCCKESSDPSSYNSSGQLMASKMDTCSSNLNNSIYKKLLT
- The following amino acid sequences were used for effector proteins:
- Human βarr2(1–351) (based on UniProt P32121, ARRB2_HUMAN): MGEKPGTRVFKKSSPNCKLTVYLGKRDFVDHLDKVDPVDGVVLVDPDYLKDRKVFVTLTCAFRYGREDLDVLGLSFRKDLFIATYQAFPPVPNPPRPPTRLQDRLLRKLGQHAHPFFFTIPQNLPCSVTLQPGPEDTGKACGVDFEIRAFCAKSLEEKSHKRNSVRLVIRKVQFAPEKPGPQPSAETTRHFLMSDRSLHLEASLDKELYYHGEPLNVNVHVTNNSTKTVKKIKVSVRQYADICLFSTAQYKCPVAQLEQDDQVSPSSTFCKVYTITPLLSDNREKRGLALDGKLKHEDTNLASSTIVKEGANKEVLGILVSYRVKVKLVVSRGGDVSVELPFVLMHPKPHD
- Human Gαq (1–354) (based on UniProt P50148, GNAQ_HUMAN): MTLESIMACCLSEEAKEARRINDEIERQLRRDKRDARRELKLLLLGTGESGKSTFIKQMRIIHGSGYSDEDKRGFTKLVYQNIFTAMQAMIRAMDTLKIPYKYEHNKAHAQLVREVDVEKVSAFENPYVDAIKSLWNDPGIQECYDRRREYQLSDSTKYYLNDLDRVADPAYLPTQQDVLRVRVPTTGIIEYPFDLQSVIFRMVDVGGQRSERRKWIHCFENVTSIMFLVALSEYDQVLVESDNENRMEESKALFRTIITYPWFQNSSVILFLNKKDLLEEKIMYSHLVDYFPEYDGPQRDAQAAREFILKMFVDLNPDSDKIIYSHFTCATDTENIRFVFAAVKDTILQLNLK
- Human Gβ1 (1–340) (UniProt P62873, GBB1_HUMAN): MSELDQLRQEAEQLKNQIRDARKACADATLSQITNNIDPVGRIQMRTRRTLRGHLAKIYAMHWGTDSRLLVSASQDGKLIIWDSYTTNKVHAIPLRSSWVMTCAYAPSGNYVACGGLDNICSIYNLKTREGNVRVSRELAGHTGYLSCCRFLDDNQIVTSSGDTTCALWDIETGQQTTTFTGHTGDVMSLSLAPDTRLFVSGACDASAKLWDVREGMCRQTFTGHESDINAICFFPNGNAFATGSDDATCRLFDLRADQELMTYSHDNIICGITSVSFSKSGRLLLAGYDDFNCNVWDALKADRAGVLAGHDNRVSCLGVTDDGMAVATGSWDSFLKIWN
- Human Gγ (1–69) (UniProt P59768, GBG2_HUMAN): MASNNTASIAQARKLVEQLKMEANIDRIKVSKAAADLMAYCEAHAKEDPLLTPVPASENPFREKKFFCA

One hundred models (20 random seeds with 5 models per seed) were built for each of the following proteins and complexes.

- Uncomplexed PAR1(22–390)
- Uncomplexed PAR1(42–390)
- Uncomplexed PAR1(47–390)
- PAR1(amino acids 42–425, containing pT410, pS412, pS413, pS418) with βarr2
- PAR1(amino acids 47–425, containing pS391, pS392, pS395, pS396, pS399, pS400, pT410, pS412, pS413, pS418) with βarr2
- PAR1(amino acids 42–425, containing pT410, pS412, pS413, pS418) with Gαq, Gβ1, Gγ2, and βarr2
- PAR1(amino acids 47–425, containing pS391, pS392, pS395, pS396, pS399, pS400, pT410, pS412, pS413, pS418) with Gαq, Gβ1, Gγ2, and βarr2

For uncomplexed receptor models, the removal of the receptor C terminus (amino acids 391–425) prevented the conformational bias due to its frequently predicted insertion into the effector binding surface and thus enabled the studies of conformational preferences of PAR1 TM domain as a function of N-terminal proteolytic cleavage. For models with βarr2, the removal of the βarr2 C terminus (amino acids 352–409) prevented the self-inhibited βarr2 conformation where its C terminus blocks the N-lobe interface and enabled studies of binding preferences for the differentially phosphorylated PAR1 C terminus.

The resulting complexes were superimposed by the receptor TM domain, visualized, and analyzed in ICM v3.9–3a (Molsoft LLC, San Diego, CA). Inter-and intramolecular distances were measured using python library *MDAnalysis*,^65,66^ 2D pseudo-color histograms were plotted with *matplotlib*.^67^ Confidence metrics (pLDDT, PAE, ipTM) were extracted from the json files generated by the AlphaFold 3 server. Average effector motif PAE values were calculated for amino acids 64–77 of βarr2 and amino acids 332–359 of Gαq. Violin plots were built in R using *ggplot2*.^68^

#### Cell culture

HUVEC-derived endothelial EA.hy926 cells (ATCC #CRL-2922) were maintained in 10% fetal bovine serum (FBS) (Gibco #10437–028) in Dulbecco’s Modified Eagle Medium (DMEM) (Corning #10–013-CV) supplemented with fresh 20% preconditioned media every 2–3 days, grown at 37°C in 8% CO_2_ and used up to passage 8.^12^ EA.hy926 cells were authenticated by short tandem repeat profiling. Primary HUVECs (Lonza #C2519A) were maintained in endothelial cell growth medium-2 (Lonza #CC-3162) and media was changed every 2 days, grown at 37°C in 5% CO_2_ and used up to passage 6. HUVEC were authenticated by expression of CD31/105 and von Willebrand Factor VIII, and assessment of acetylated low-density lipoprotein uptake. HEK293A parental cells, HEK293A CRISPR-Cas9 β-arrestin-1,2 KO cells, and HEK293A GRK 2,3,5,6 KO cells were obtained from Dr. Asuka Inoue (Tohoku University) and verified by restriction enzyme-based genotyping and immunoblot.^37,62^ Cells were maintained in 10% FBS in DMEM supplemented with fresh media every 2 to 3 days, grown at 37°C in 5% CO_2_ and used up to passage 10. HeLa-PAR1 cells were generated as previously described^54^ and grown in DMEM supplemented with 10% FBS and 250 μg/mL hygromycin B and used up to passage 10. All cell lines were routinely monitored for mycoplasma using Venor GeM Mycoplasma detection kit (Sigma-Aldrich) per the manufacturer’s instructions.

#### Expression vectors

GRK5 WT, K215R, and 4A mutant pcDNA3 plasmids^32,63^ were from Dr. Philip B. Wedegaertner (Thomas Jefferson University). PAR1-YFP^64^ was obtained from Dr. Jean-Philippe Pin (University of Montpellier). EPCR-Halo, RLuc-βarr2, PAR1 C terminus phospho-site mutants 0P, hel8, dP2, and dP3 fused to YFP were generated by Gibson assembly homologous recombination (New England Biolabs, Gibson Assembly Master Mix #E2611L) followed by whole plasmid sequencing. The Nluc-βarr1 and -βarr2 WT and dFLR mutant plasmids and Nluc-βarr2 FlAsH variants plasmids^39^ were from Dr. Carsten Hoffmann (University Hospital Jena).

#### qRT-PCR

Endothelial cells were seeded in a 6-well plate at 3.2 × 10^5^ cells per well and grown to confluency. RNA was extracted using the Direct-zol RNA Miniprep Plus Kit (Zymo Research #R2072) and used to generate complementary DNA (cDNA) according to the manufacturer’s instructions. RNA was quantified and cDNA synthesized from 1 μg RNA using SuperScript IV VILO Master Mix with ezDNase enzyme kit (Thermo Fisher Scientific #111766050). qRT-PCR was performed with TaqMan Fast Advanced Master Mix (Thermo Fisher Scientific #4444964) and TaqMan Gene Expression Probes GRK2 (#Hs00176395), GRK3 (#Hs00178266), GRK5 (#Hs00992173), GRK6 (#Hs00357776), and 18S (#Hs03003631_g1) using a QuantStudio 3 Real-Time PCR System (Thermo Fisher Scientific). GRK mRNA transcript levels were normalized to 18S expression. The differences in expression relative to GRK5 were then determined using the 2-^ΔΔ^Ct method. Control reactions without cDNA for each probe were conducted in every assay to ensure specificity of the reactions. The data are expressed as a fraction of GRK5 mRNA transcript expression.

#### siRNA transfections

Endothelial cells were seeded in a 12-well plate at 2.5 × 10^5^ cells per well, grown overnight, and transfected with 25 nM GRK2 #1 siRNA (5′-CCGGGAGATCTTCGACTCATA-3′) (Qiagen #SI00287378), 25 nM GRK5 #5 siRNA (5′-AGCGTCATAACTAGAACTGAA-3′) (Qiagen #SI00287770) or 25 nM non-specific siRNA (Qiagen #1027281 AllStars Negative Control siRNA) using the TransIT-X2 System (Mirus, #MIR 6000) according to the manufacturer’s instructions. Whole cell lysates were collected 48 h post transfection.

#### Signaling assays

After siRNA transfection, endothelial cells were serum-starved overnight in 0.4% FBS-DMEM. Cells were then incubated in serum-free DMEM containing 10 mM HEPES, 1 mM CaCl_2_, and 1 mg/mL bovine serum albumin (BSA) for 1 h prior to incubation with thrombin (10 nM) or APC (20 nM) at 37°C. After agonist stimulation, cells were lysed in 2x Laemmli sample buffer (LSB) containing 200 mM dithiothreitol (DTT), heated for 5 min at 95°C, resolved by SDS-PAGE and immunoblotted.

HEK293 cells were harvested with Triton lysis buffer (50 mM Tris pH 7.4, 100 mM NaCl, 5 mM EDTA, 1% v/v Triton X-100, 50 mM NaF, and 10 mm NaPP) containing protease inhibitors including 1 μg/mL of leupeptin, aprotinin, trypsin protease inhibitor or pepstatin, benzamidine 100 μg/mL, PMSF 100 μg/mL and quantified by bicinchoninic acid (BCA) protein assay (Thermo Fisher Scientific, #A55860). Equivalent amounts of cell lysates were diluted in 2x LSB with 200 mM DTT, heated for 5 min at 95°C, resolved by SDS-PAGE, transferred to the PVDF membrane and immunoblotted. Immunoblots were quantified by densitometry using NIH ImageJ software.^69^

#### Immunofluorescence confocal microscopy

HeLa cells were seeded at 1.0 × 10^5^ per coverslip transfected with GRK5 WT, K215R, 4A mutant or pcDNA3. Cells were fixed with 4% paraformaldehyde, permeabilized with 0.1% Triton X-100 and labeled with monoclonal anti-GRK5 antibody (at 1: 500) for 1 h on ice. Cells were then incubated with anti-mouse Alexa 488 diluted to 1:750 and DAPI at 1 mg/mL in 0.03% BSA, 0.01% Triton X-100, and 0.01% normal goat serum at room temperature for 1 h. Slides were mounted using ProLong Gold Antifade Mountant (Invitrogen #P10144). Confocal images were acquired with an Olympus IX81 spinning-disk microscope equipped with a CoolSNAP HQ2 CCD camera (Andor) and 63× Plan Apo objective (1.4 NA) with appropriate excitation-emission filters using Metamorph software. Line scan analysis was performed using NIH ImageJ software.

#### BRET assays

HEK293 cells were seeded in a 6-well plate at 4.5 × 10^5^ cells per well, grown overnight, and transfected with PAR1 WT, 0P, dP2 or dP3 mutants fused to YFP, APC co-receptor EPCR-Halo and, RLuc-βarr2 wildtype or pcDNA3 diluted in Opti-Mem and transfected with polyethylenimine (PEI) at a 1:3 ratio. In other BRET experiments, GRK5 WT, 4A or K215R and Nluc-βarr2- or Nluc-βarr1WT and dLR mutants were combined with wildtype PAR1-YFP and EPCR-Halo diluted in Opti-Mem and transfected with PEI. After 24 h of transfection, cells were collected and re-seeded into poly-D-lysine coated 96-well plate at 3 ×10^4^ cells per well and grown overnight. Cells were washed with PBS and serum-starved for 1 h using a 1:1 equal mixture of DMEM without phenol red (Gibco #31053–028) combined with PBS. In some experiments, cells were pretreated with 10 μM vorapaxar for 30 min during starvation. After starvation, cells were preincubated with 5 μM of Coelenterazine H for 5 min followed by the addition of 1 nM thrombin or 20 nM APC and BRET measurements were taken at 37°C over time. All BRET measurements were performed with a Berthold TriStar LB941 multimode plate reader using MikroWIN 2010 software (Berthold Biotechnologies) using two filter settings: 480 nm for Rluc and 530 nm for YFP. The BRET signal was calculated as the emission at 530 nm divided by the emission at 480 nm. The BRET signals were normalized to basal BRET ratios and expressed as the percent over basal.

FlAsH BRET assays were performed in HEK293 cells, seeded, grown and transfected with wildtype PAR1-YFP, EPCR-Halo and the Nluc-βarr2 N-domain or C-domain FlAsH constructs^39^ as described in BRET assays above. Cells were serum starved and incubated with 2 mM FlAsH-EDT2 (Thermo Fisher Scientific #T34561) for 30 min, washed with BAL (25 mM dimercaprol in 10 mM HEPES buffer, pH 7.3) for 5 min, and serum starved for an additional 30 min. After starvation, cells were preincubated with 5 μM of Coelenterazine H for 3 min followed by the addition of 1 nM thrombin or 20 nM APC and BRET measurements were taken for 5 min at 37°C and net BRET determined as described above.

#### CELL SURFACE ELISA

HEK293 GRK KO cells seeded at 1 ×10^5^ cells per well in 12-well plates were grown overnight and transfected with 400 ng PAR1-YFP WT, mutants or pBJ vector control together with 200 ng EPCR-Halo, 100 ng RLuc-βarr2 and 50 ng GRK5 WT. After 48 h, cells were fixed with 4% PFA, washed, incubated with anti-PAR1 polyclonal antibody (1:200) or pre-immune serum (1:100) for 1 h at room temperature followed by incubation with secondary antibody for 1 h. Cells were washed and then incubated with 1-Step (2,2′-Azinobis [3-ethylbenzothiazoline-6-sulfonic acid]-diammonium salt) ABTS substrate solution (Thermo Fisher Scientific, #37615) for 10–15 min at room temperature, an aliquot was removed and the absorbance measured at 405 nm using a Molecular Devices SpectraMax ABS Plus microplate reader as described.^70^

### QUANTIFICATION AND STATISTICAL ANALYSIS

Student’s *t* test was used for comparison of two groups and for comparison of multiple groups, one-way or two-way ANOVA was used, with repeated measures followed by post-hoc test with multiple comparisons corrections. *p* values ≤0.05 were considered to differ significantly where *p < 0.05, **p < 0.01, ***p < 0.001 and ****p < 0.0001. The data are presented as the mean ± standard deviation (S.D.). The number of experimental replicates is indicated in the figure legends. Statistical analysis and generation of graphs were conducted with Prism 10.2 statistical software (GraphPad Software, La Jolla, CA).

## Supplementary Material

1

SUPPLEMENTAL INFORMATION

Supplemental information can be found online at https://doi.org/10.1016/j.celrep.2026.117041.

## Figures and Tables

**KEY RESOURCES TABLE:** 

REAGENT or RESOURCE	SOURCE	IDENTIFIER

Antibodies		

Mouse anti-GRK4-6 A16/17	Millipore	Cat #05-466; RRID:AB_11213523
Mouse anti-GRK2/3 C5/1.1	Millipore	Cat #05-465; RRID:AB_11213371
Mouse anti-Renilla Luciferase	Millipore	Cat #MAB4400; RRID:AB_95116
Mouse anti-β-Actin AC-74	Sigma-Aldrich	Cat #A5316; RRID:AB_476743
Rabbit polyclonal anti-GRK5	Thermo Fisher Scientific	Cat #PA5-96262; RRID:AB_2808064
Rabbit anti-phospho-p38 MAPK T180/Y182	Cell Signaling Technology	Cat #4511; RRID:AB_2139682
Rabbit anti-p38 MAPK polyclonal	Cell Signaling Technology	Cat #9212; RRID:AB_330713
Rabbit monoclonal anti-phospho-Akt-S473 D9E	Cell Signaling Technology	Cat #4060; RRID:AB_2315049
Rabbit anti-Akt polyclonal	Cell Signaling Technology	Cat #9272; RRID:AB_329827
Rat anti-GFP c3H9	ChromoTek	Cat #3h9; RRID:AB_10773374
Rabbit anti-PAR1 polyclonal antibody C5433	Paing et al.^55^	N/A
Goat anti-mouse horseradish peroxidase (HRP)	Bio-Rad	Cat #170-6516; RRID:AB_11125547
Goat anti-rabbit horseradish peroxidase (HRP)	Bio-Rad	Cat #170-6515; RRID:AB_11125142
Goat anti-mouse cross absorbed Alexa Fluor 488	Thermo Fisher Scientific	Cat #A-11001; RRID:AB_2534069

Chemicals, peptides, and recombinant proteins		

DAPI (4',6-diamidino-2-phenylindole)	Thermo Fisher Scientific	Cat #D-1306
α-thrombin	Enzyme Research Laboratories	Cat #HT 1002a
Activated Protein C	Prolytix	Cat #HCAPC-0080
Vorapaxar	Axon MedChem	Cat #1755
Coelentrazine H	Biotium	Cat #1011-1
ProLong Gold Antifade Mountant	Invitrogen	Cat #P10144
Endothelial cell growth medium	Lonza	Cat #CC-3162
1-Step ABTS (2,2'-azino-bis(3-ethylbenzothiazoline-6-sulfonici acid)) substrate solution	Thermo Fisher Scientific	Cat #37615
TransIT-X2 System	Mirus	Cat #MIR 6000
Fetal bovine serum	Gibco	Cat #10437-028
Dulbecco's Modified Eagle Medium	Corning	Cat # 10-013-CV
DMEM without phenol red	Gibco	Cat #31053-028

Critical commercial assays		

Pierce bicinchoninic acid (BCA) protein assay	ThermoFisher Scientific	#A55860
Direct-zol RNA Miniprep Plus Kit	Zymo Research	#R2072
SuperScript IV VILO Master Mix with ezDNase enzyme kit	Thermo Fisher Scientific	#111766050
TaqMan Fast Advanced Master Mix	Thermo Fisher Scientific	#4444964
TC-FlAsH In-Cell Tetracysteine Tag Detection Kit	Thermo Fisher Scientific	#T34561
Gibson Assembly Master Mix	New England Biolabs	#E2611L

Deposited data		

Coordinates of PAR1 AF3 model ensembles	Zenodo	https://doi.org/10.5281/zenodo.18816262

Experimental models: Cell lines		

HUVEC	Lonza	#C2519A
HUVEC-derived endothelial EA.hy926 cells	ATCC	#CRL-2922
HEK293A parental cells	O'Hayre et al.^37^; Saito et al.^62^	N/A
HEK293A CRISPR-Cas9 β-arrestin-1,2 KO cells	O'Hayre et al.^37^; Saito et al.^62^	N/A
HEK293A GRK 2,3,5,6 KO cells	O'Hayre et al.^37^; Saito et al.^62^	N/A
HeLa-PAR1 cells	Paing et al.^54^	N/A

Oligonucleotides		

GRK2 #1 siRNA 5'-CCGGGAGATCTTCGACTCATA-3'	Qiagen	#SI00287378
GRK5 #5 siRNA 5'-AGCGTCATAACTAGAACTGAA-3'	Qiagen	#SI00287770
AllStars non-specific siRNA	Qiagen	#1027281
GRK2 Taqman probe FAM-MGB	ThermoFisher Scientific	#Hs00176395
GRK3 Taqman probe FAM-MGB	ThermoFisher Scientific	#Hs00178266
GRK5 Taqman probe FAM-MGB	ThermoFisher Scientific	#Hs00992173
GRK6 Taqman probe FAM-MGB	ThermoFisher Scientific	#Hs00357776
18S Taqman probe VIC-MGB	ThermoFisher Scientific	#Hs03003631_g1

Recombinant DNA		

GRK5 wildtype (WT) pcDNA3 plasmid	Thiyagarajan et al.^32^; Xu et al.^63^	N/A
GRK5 K214R mutant pcDNA3 plasmid	Thiyagarajan et al.^32^; Xu et al.^63^	N/A
GRK5 4A mutant pcDNA3 plasmid	Thiyagarajan et al.^32^; Xu et al.^63^	N/A
PAR1 WT-YFP in pRK6 plasmid	Ayoub et al.^64^	N/A
PAR1 0P mutant-YFP pRK6 plasmid	This paper	N/A
PAR1 hel8 mutant YFP pRK6 plasmid	This paper	N/A
PAR1 dP2 mutant-YFP pRK6 plasmid	This paper	N/A
PAR1 dP3 mutant-YFP pRK6 plasmid	This paper	N/A
EPCR-Halo pcDNA3	This paper	N/A
Rluc-βarr2 WT pcDNA3	This paper	N/A
Nluc-βarr2 WT pcDNA3	Haider et al.^39^	N/A
Nluc-βarr2 dFLR pcDNA3	Haider et al.^39^	N/A
Nluc-βarr1 WT pcDNA3	Haider et al.^39^	N/A
Nluc-βarr1 dFLR pcDNA3	Haider et al.^39^	N/A
βarr2-FlAsH1-Nluc pcDNA3	Haider et al.^39^	N/A
βarr2-FlAsH2-Nluc pcDNA3	Haider et al.^39^	N/A
βarr2-FlAsH3-Nluc pcDNA3	Haider et al.^39^	N/A
βarr2-FlAsH4-Nluc pcDNA3	Haider et al.^39^	N/A
βarr2-FlAsH5-Nluc pcDNA3	Haider et al.^39^	N/A
βarr2-FlAsH7-Nluc pcDNA3	Haider et al.^39^	N/A
βarr2-FlAsH9-Nluc pcDNA3	Haider et al.^39^	N/A
βarr2-FlAsH10-Nluc pcDNA3	Haider et al.^39^	N/A

Software and algorithms		

AlphaFold Server (powered by AlphaFold 3), Google DeepMind, accessed [February 2025 through August 2025]	Abrambson et al.^24^	https://alphafoldserver.com/
ICM Pro 3.9-4a (Molsoft LLC, San Diego, CA)	N/A	https://www.molsoft.com/icm_pro.html
python, MDAnalysis 2.7.0	Michaud-Agrawal et al.^65^; Gowers et al.^66^	https://www.mdanalysis.org/
python, DESRES msys 1.7.359	N/A	https://msys.readthedocs.io/en/latest/
python, matplotlib 3.10.0	Hunter et al.^67^	https://ggplot2.tidyverse.org/
Chimera X for the βarr2 FlAsH construct illustrations	N/A	https://www.rbvi.ucsf.edu/chimerax/docs/credits.html
Violin plots were built in R using ggplot2	Wickham^68^	https://link.springer.com/book/10.1007/978-3-319-24277-4
ImageJ	Schneider et al.^69^	https://imagej.net/ij/; RRID:SCR_003070
GraphPad Prism 10.2, GraphPad Software, La Jolla, USA	N/A	https://www.graphpad.com/; RRID:SCR_002798
BioRender software	N/A	https://www.biorender.com; RRID:SCR_018361
Adobe Illustrator 29.5.1	N/A	http://www.adobe.com/products/illustrator.html; RRID:SCR_010279
Metamorph software	Molecular Devices, Sunnyvale, CA	RRID: SCR_002368
MikroWin software 5.24	Labsis Laborsysteme GmbH	N/A

## Highlights

- AlphaFold 3 modeling explains the basis of PAR1 signaling bias to thrombin and APC
- GRK5 membrane anchoring differentially regulates thrombin and APC signaling bias
- PAR1 phosphorylation and **β**-arrestin binding distinguish thrombin versus APC signaling
- Unique **β**-arrestin conformations underlie thrombin versus APC bias signaling
