# Supplementary material for: Signaling bias of the protease-activated receptor-1 is dictated by distinct GRK5 and β-arrestin-2 determinants
Source: Cell Rep. Author manuscript; Available in PMC 2026 Apr 18. (PMC13091039; doi:10.1016/j.celrep.2026.117041)
Supplement: 1 [file NIHMS2160121-supplement-1.pdf]

Cell Reports, Volume *45*

Supplemental information

Signaling bias of the protease-activated receptor-1 is dictated by distinct GRK5 and β-arrestin-2 determinants

Monica L. Gonzalez Ramirez, Lennis B. Orduña-Castillo, Carolyne Bardeleben, Huaping Qin, Ying Lin, Cierra A. Birch, Irina Kufareva, and JoAnn Trejo

Supplementary information

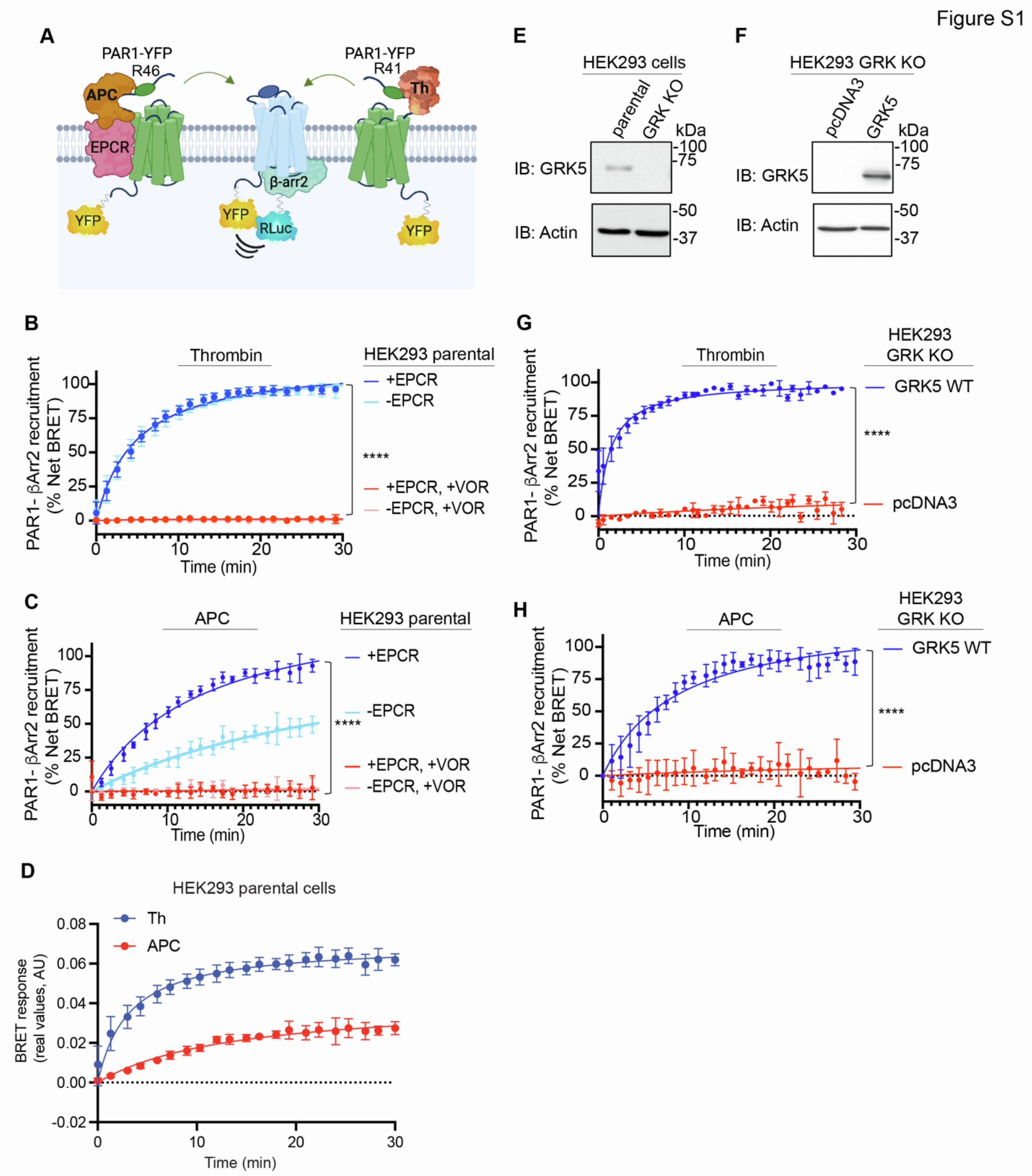

Figure S1. **Thrombin- versus APC-activated PAR1 induced βarr2 recruitment in HEK293 parental cells, related to Figure 3**

A, Cartoon of BRET assay illustrating APC/EPCR- and thrombin-induced βarr2 recruitment. HEK293 cells expressing PAR1-YFP, EPCR-Halo and Rluc-βarr2, treated with vorapaxar or DMSO vehicle were stimulated with 1 nM thrombin (B) or 20 nM APC (C) and βarr2 recruitment was determined by BRET.

Data (mean ± S.D., n=3) was analyzed by one-way ANOVA followed by Tukey's multiple comparisons test (****, $p < 0.0001$). D, HEK293 parental cells transfected with PAR1-YFP, EPCR-Halo and Rluc-βarr2, pretreated with vorapaxar or DMSO vehicle, and stimulated with 1 nM thrombin or 20 nM APC, after which βarr2 recruitment to PAR1 was determined by BRET. Non-normalized BRETnet responses to thrombin and APC are graphed as mean ± S.D. from three independent experiments over a 30 min time-course. E and F, HEK293 cells transfected with GRK5 WT or pcDNA3 were immunoblotted as indicated. HEK293 GRK KO cells transfected with PAR1-YFP, EPCR-Halo, Rluc-βarr2 and GRK5 WT or pcDNA3 were stimulated with 1 nM thrombin (G) or 20 nM APC (H) and βarr2 recruitment determined by BRET. Data (mean ± S.D., n=3) was analyzed by Student's *t* test (****, $p < 0.0001$).

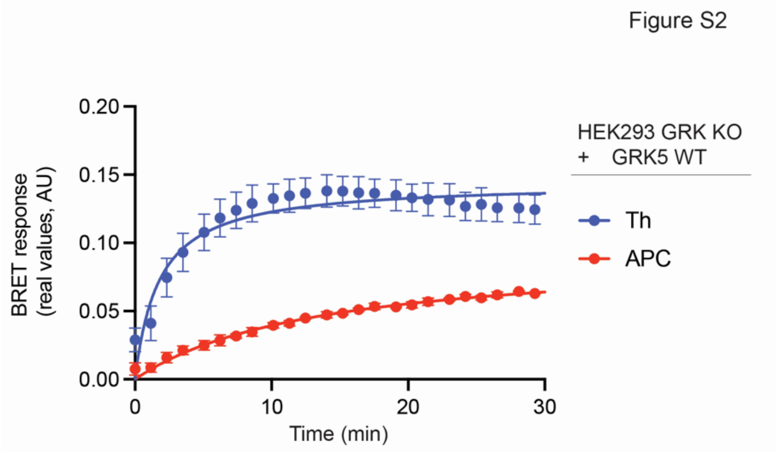

Figure S2. **Thrombin- *versus* APC-induced βarr2 recruitment to PAR1 in HEK293 GRK KO cells expressing GRK5 wildtype, related to Figure 3**

HEK293 GRK KO cells co-expressing PAR1-YFP, EPCR-Halo and Rluc-βarr2 with GRK5 wildtype (WT) were stimulated with 1 nM thrombin (Th) or 20 nM APC and βarr2 recruitment to PAR1 was determined by BRET. Non-normalized BRETnet responses for Th and APC are graphed as the mean ± S.D. from three independent experiments over a 30 min time-course. Normalized data is shown in Figure 3C and D (main text).

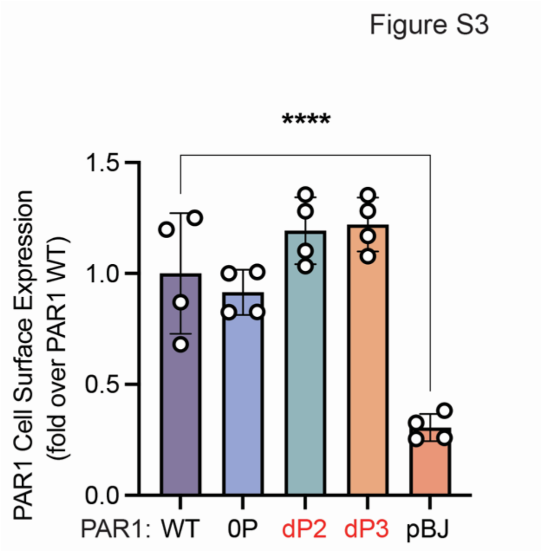

Figure S3. **Cell surface expression of PAR1-YFP wildtype and phospho-site mutants, related to Figure 4**

HEK293 GRK KO cells transfected with PAR1 wildtype (WT), 0P, dP2, dP3 mutants or pBJ vector along with EPCR-Halo, RLuc-βarr2 and GRK5 WT were fixed and processed for cell surface ELISA. Data mean ± S.D.) from four independent were analyzed by one-way ANOVA, ****$p < 0.0001$.

Figure S4

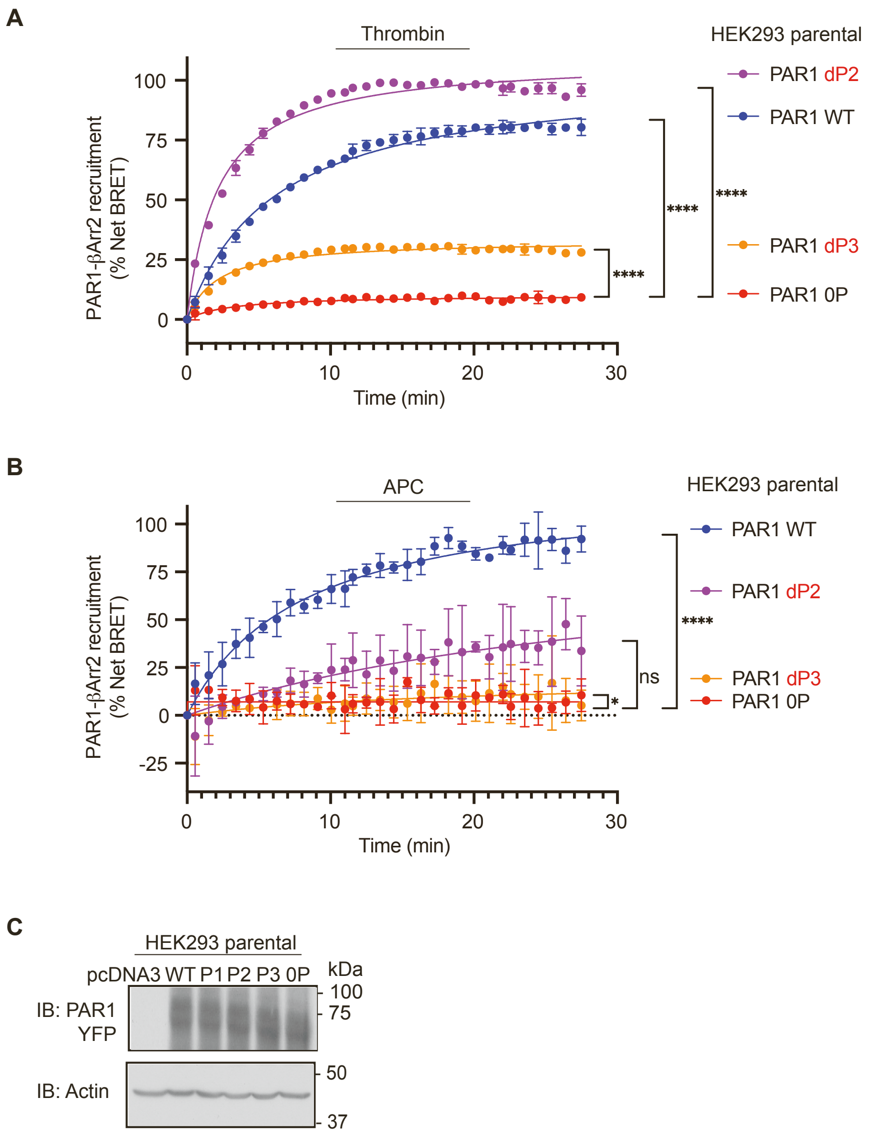

Figure S4. **Thrombin- *versus* APC-induced βarr2 recruitment require distinct PAR1 C-terminal phosphorylation sites in HEK293 parental cells, related to Figure 4**

A and B, HEK293 parental cells were transfected with PAR1-YFP wildtype (WT) or 0P, dP2 or dP3 mutants together with EPCR-Halo and Rluc-βarr2 were stimulated with 1 nM thrombin or 20 nM APC and βarr2 recruitment was determined by BRET. C**,** HEK293 parental cell lysates were immunoblotted as indicated. Actin was used as a loading control. The data (mean ± S.D.) from three independent replicates was analyzed by one-way ANOVA followed by Tukey's multiple comparisons test (****, $p < 0.0001$; ns = not significant).

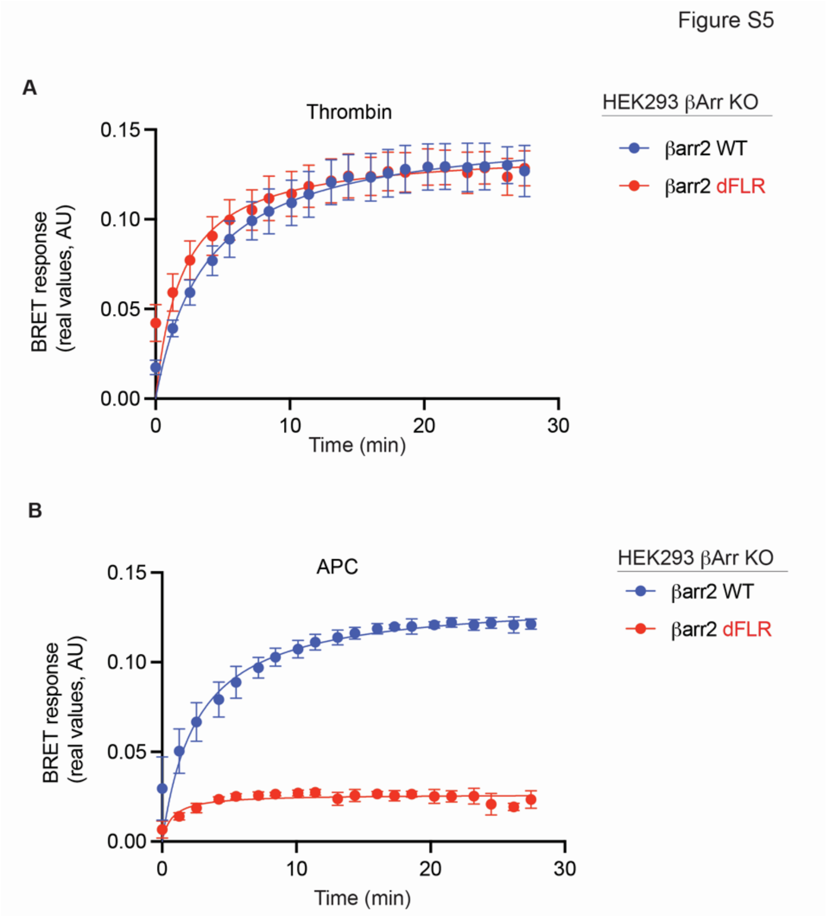

Figure S5. **Thrombin- *versus* APC-induced βarr2 recruitment to PAR1 in HEK293 βarr1,2 KO cells expressing βarr2 wildtype or finger loop finger mutant, related to Figure 5**

HEK293 CRISPR-Cas9 βarr1,2 KO cells transfected with PAR1-YFP, EPCR-Halo and either Nluc-βarr2 WT or Nluc-βarr2 finger loop region (dFLR) mutant were stimulated with either (A) 1 nM thrombin (Th) or (B) 20 nM APC and βarr2 recruitment was determined by BRET. Non-normalized BRETnet responses for Th and APC are graphed as the mean ± S.D. from three independent experiments over a 30 min time-course. Normalized data is shown in Figure 5D and E (main text).

Figure S6

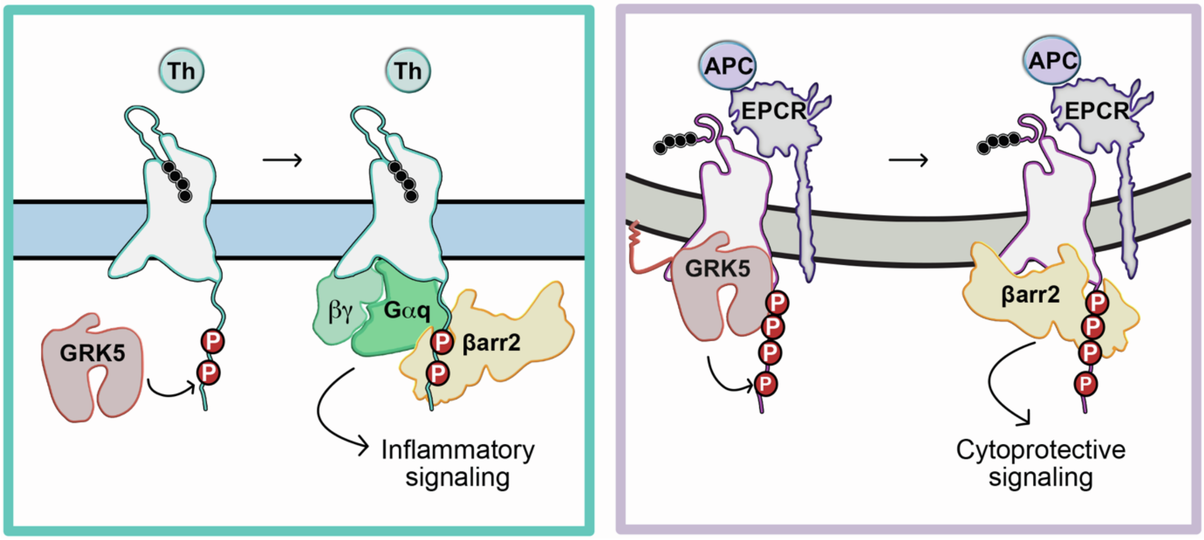

Figure S6. **Model of PAR1 signaling bias**

PAR1 displays biased signaling in response to thrombin (G protein and βarr) and APC (βarr only). APC/PAR1 and not Th/PAR1 signaling occurs in caveolae microdomains. Th- and APC-induced PAR1 signaling are both regulated by GRK5 but differentially dependent on GRK5 membrane anchoring and C-terminus phosphorylation. In addition, Th- and APC-activated PAR1 engage βarr2 through different binding modes, resulting in distinct βarr2 conformations that facilitate APC cytoprotective signaling *versus* thrombin inflammatory signaling.
